# Csf1r⁻ macrophages govern the second wave of neutrophils for bone repair

**DOI:** 10.21203/rs.3.rs-6695752/v1

**Published:** 2025-05-22

**Authors:** Yasuhiro Kobayashi, Zhifeng He, Linan Shi, Yuko Nakamichi, Kohei Murakami, Toshide Mizoguchi, Yuki Matsushita, Hirotaka Matsumoto, Shinichirou Ito, Shunsuke Uehara, Rina Iwamoto, Takumi Takahashi, Zizhao Tian, Toru Hiraga Hiraga, Ruoxuan Li, Masataka Kasahara, Kazuo Okamoto, Hiroshi Takayanagi, Martin Matzuk, Nobuyuki Udagawa

## Abstract

Macrophages play a pivotal role in tissue regeneration, including bone healing; however, the mechanisms and cell types through which they establish regenerative niches are not yet fully understood. Here, we identified a distinct subset of osteodirective macrophages (OdMacs), characterized by the expression profile F4/80⁺ Csf1r⁻ Cx3cr1hi Ccr2hi, which contribute to the formation of bone regenerative niches following injury. Histological analysis and FACS analysis, combined with transcriptomic profiling (RNA-seq and scRNA-seq), revealed that OdMacs facilitate the recruitment of activin A-expressing preneutrophils (preNeu) to sites of bone injury. This recruitment enhances the proliferation and osteogenic differentiation of LepR⁺ bone marrow stromal cells (BMSCs), thereby promoting bone regeneration. Furthermore, genetic studies using Axin2-Cre; tdTomato, Inhba, and Wls conditional knockout mice underscored the essential roles of Wnt signaling in BMSCs and preNeu-derived activin A in orchestrating this regenerative process. The OdMac-regulated regenerative niche represents a promising therapeutic target for regenerative medicine.

### Introduction

The skeletal and immune systems are considered to interact because they have been co-developed when terrestrial organisms firstly appeared [1]. Fracture healing is a multi-step process, progressing from hematoma formation to bone marrow formation [2]. Neutrophils (Neu) and Mφs are recruited to the injury sites and play key roles in the early steps of the healing such as inflammation. Additionally, Neu have been shown to have opposing roles in fracture healing. On one hand, they inhibit bone formation by inducing Neu extracellular trap formation [3] or by directly interacting with osteoblasts [4]. On the other hand, Neu promote the recruitment of BMSC and initiate bone repair [5]. This dual role suggests that Neu consist of heterogenous cell populations whose osteogenic functions may change in response to microenvironment factors during fracture healing.

Mφs are also involved in the fracture healing because they are present throughout the entire bone regeneration process [6] and promote fracture healings as well as bone remodeling [7, 8]. In fact, bone-resident Mφs, reside in the vicinity of osteoblasts to regulate bone remodeling. These cells have been characterized as F4/80(+) Mac-2$^{(-)/\ low}$ TRAP(–) subset, though they have not been fully identified [9]. Mφs are not only able to remove cell debris and eliminate pathogens during wound repair but also modulate the recruitment of BMSCs and vascular cells from both bone marrow and periosteum [10-12]. However, the molecular mechanisms by which different subsets of Mφs and Neu interact with their surroundings to facilitate fracture healings remain poorly understood.

Here, we demonstrate that F4/80(+) Csf1r(–) Cx3cr1$^{hi}$ Ccr2$^{hi}$ cells rather than classical Csf1r(+) monocyte-derived Mφs, are highly abundant in the early stages at bone injury sites. Depletion of this Mφ subset via clodronate liposome administration results in impaired osteogenesis at bone injury sites and compromised activation of LepR(+) BMSCs during the inflammatory phase. Additionally, we have discovered that this novel osteodirective Mφ subset as a key regulator of bone regeneration, acting in coordination with activin A-producing preNeu. The presence of Mφs ensures a sufficient supply of activin A, which promotes the expansion of BMSCs at the site of bone injury. This, in turn, activates Wnt signaling to drive regenerative osteogenesis. These findings highlight the critical role of this unique Mφ subset in fracture healing and suggest potential new therapeutic strategies for enhancing bone regeneration.

### Results

#### F4/80(+)Csf1r(–) phagocytes targeted by clodronate liposome are indispensable for bone repair

To investigate effects of Mφ depletion on cortical bone regeneration and bone induction in bone marrow, we administered clodronate-liposome (Clo-lip; [13]) intraperitoneally every other day into mice that had undergone bone injury surgery (Fig. 1a). Immunofluorescent analysis revealed a marked reduction of F4/80(+) Mφs at the injury site of Clo-lip-treated mice (Fig. 1b).

Micro-CT analysis showed that the induction of bone was hindered in injured areas of the Mφ-depleted mice compared to mice treated with control liposome (Ctrl-lip) (Fig. 1c). We then investigated the impact of Mφ depletion on the bone injury-induced osteoblast differentiation using immunofluorescent techniques and *Col1(2.3)*-GFP mice, in which osteoblastic cells show a green fluorescent signal [14, 15]. The number of *Col1(2.3)*-GFP(+) (Fig. S1a) and osteocalcin(+) osteoblasts (Fig. S1b) decreased at the injury site of Mφ-depleted mice at 7 days post-injury (7 dpi). These findings indicate that F4/80(+) Mφs play a crucial role in osteogenesis at the injury sites.

To confirm roles of the Mφ depletion in bone regeneration and induction, we analyzed bone injury sites in other Mφ depletion models; one treated with anti-Csf1r neutralizing antibodies (clone AFS98, Fig 1d) and the other utilizing *Csf1r*MerCreMer; *Csf1r*-floxed mice (Fig 1g). Although the administration of AFS98 and tamoxifen markedly depleted F4/80(+) Mφs — particularly F4/80(+) Csf1r(+) Mφs — at bone injury sites from each model (Fig 1e, Fig S1c-j), bone induction remained unchanged in both models (Fig 1f, h). Unlike the Clo-lip treatment (Fig. S1c, e), these two models did not exhibit a decrease in number of F4/80(+) Csf1r(–) cell fraction by histological assessment (Fig S1d, f, g-j). This suggests that F4/80(+) Csf1r(–), but not F4/80(+) Csf1r(+) cell fraction may play critical roles in bone regeneration and induction. Flow cytometry analysis using F4/80 and Csf1r antibodies further confirmed that Clo-lip administration significantly reduced F4/80(+) Csf1r(–) cell fraction compared to each control experiment (Fig. 2a). In contrast, AFS98 eliminated most of F4/80(+) Csf1r(+) cell fraction, but not F4/80(+) Csf1r(–) one (Fig. 2a). Since Mφs exhibit high phagocytic activity [16], we next tested whether F4/80(+) Csf1r(–) cell fraction has a high capacity for Clo-lip uptake. More than half of cells that internalized DiI-liposomes expressed F4/80, but not Csf1r (Fig. 2b). Histological analysis of bone injury sites also demonstrated that F4/80(+) cells exhibited significantly higher uptake of DiI-labeled liposomes compared to Csf1r(+) cells (Fig. 2c). These findings suggest that F4/80(+) Csf1r(–) cell fraction is the primary targets of Clo-lip and play a crucial rule in bone regeneration and induction.

The immunofluorescent staining using F4/80 and Csf1r antibodies showed that the F4/80(+)Csf1r(–) cells (red cells, yellow arrowheads) were highly abundant at bone injury sites during the first week (3, 4 and 5 dpi) compared with F4/80(+)Csf1r(+) one (yellow cells, white arrowheads Fig. 2d, Fig. S1k and i). This strongly suggests that F4/80(+)Csf1r(–) cells contribute to bone regeneration and induction in early healing phase. Clo-lip, AFS98, and the deletion of the *Csf1r* gene impair osteoclastogenesis [17-20]. Osteoclasts are important for bone remodeling after callus formation [9]. AFS98 and the *Csf1r* gene deletion markedly decreased osteoclast number, while Clo-lip administration had no significant effect, suggesting that osteoclasts are not largely involved in bone induction although we cannot rule out the possibility that impaired osteoclastogenesis led to increased newly formed bone in AFS98-treated and *Csf1r* cKO mice (Fig. S1m-o). Together, these results indicate that F4/80(+)Csf1r(–) cell fraction is indispensable for new bone formation during the early phase in bone repair (Fig. S2p).

BMSCs expressing leptin receptor (LepR) differentiate into osteoblasts during the fracture repair process [21]. Therefore, we performed a cell lineage analysis using *LepR*-Cre; Rosa26 tdTomato (R26-Tomato) mice in a bone injury model. *LepR*-Cre-labeled Tomato(+) cells [*LepR* Tom(+) cells] progressively increased in number and migrated toward the central area of the injury site from the intact bone marrows at 7 dpi (Fig. S2a). We observed a simultaneous expansion of *LepR* Tom(+) cells alongside F4/80(+) ones, with both cell types found in close proximity, suggesting that they may interact with each other (Fig. S2b). Indeed, the injection of Clo-lip resulted a notable decline in *LepR* Tom(+) cell number at the injury sites at 4 dpi, coinciding with the Mφ depletion (Fig. S2c). Immunostaining further revealed that *LepR* Tom(+) cells proliferated significantly at the injury sites at 4 dpi, whereas Mφ depletion markedly reduced their proliferation (Fig. S2d and e). Additionally, the expression of the osteoblastic marker gene Sp7 was significantly upregulated at 4 dpi in Ctrl-lip-treated mice but was suppressed in Clo-lip-treated mice (Fig. S2f). These findings suggest that F4/80(+) Csf1r(–) cell fraction promotes the proliferation and differentiation of *LepR* Tomato(+) stromal cells during bone regeneration.

To understand the mechanism by which Csf1r(–) cell fraction promote stromal cell activation, we performed RNA sequencing analysis of Csf1r(–) cell fraction compared to Csf1r(+) one. We focused on secreted proteins and generated a heatmap displaying the top 20 genes with significant fold change in Csf1r(–) cell fraction (Fig. 2e). Among these genes, *Inhba* (encoding activin beta A chain) caught our attention because it was expressed in immune cells and belongs to the TGF-β superfamily, reportedly to promote bone healing [22]. Neither *Inhbb* nor *Inha* mRNA was detected in F4/80(+) cells (Fig. 2f). The expression of *Inhba* mRNA was increased at bone injury sites but was suppressed by the Clo-lip treatment (Fig. 2g), suggesting that *Inhba* may be expressed in Csf1r(–) cell fraction or indirectly regulated by their depletion. In addition to the transcriptome analysis, flow cytometry analysis revealed that the F4/80(+) Csf1r(–) cell fraction is not a homogeneous population (Fig. 2e, h).

**PreNeu is the main source of activin A to promote bone repair**

To investigate whether a new subset of Mφs expresses activin A, we performed single cell RNA-seq analysis on F4/80(+) Csf1r(+), F4/80(+) Csf1r(–), and F4/80(–) Csf1r(+) cell fractions isolated from both the bone injury site (3 dpi) and the uninjured site. Integrated analyses of data from the both sites identified sixteen clusters, including Mφs, Neu lineage cells, lymphocytes, and small populations of other immune cells (Fig. 3a and Fig. S3a). None of the identified Mφ subsets exhibited high expression of *Inhba*. Instead, *Inhba* was predominantly expressed in the preNeu clusters which exhibiting high expression of *Ly6g* but lacked expression of a mature Neu marker *Cxcr2* (Fig. 3b).

We next examined cell kinetics of Neu and preNeu during bone healing. Immunofluorescent studies revealed that Ly6g(+) Ki67(–) mature Neu were present at the injury site 1 dpi, consistent with previous findings [23] (Fig. 3c). By 3 dpi, most mature Neu had disappeared from the central region of the injury site. Notably, Ly6g(+) Ki67(+) preNeu increased at the boundary area, where F4/80(+) macrophages and *LepR* Tom(+) stromal cells were accumulated (Fig. 3d, Fig. S2b). Flow cytometry analysis confirmed a significant increase in the percentage of Ly6g(+) Cxcr2(–) preNeu at 3 dpi (Fig. 3e).

To clarify the importance of preNeu-derived activin A during bone regeneration and induction following bone injury, we compared bone induction in *Vav1*-Cre; *Inhba*-floxed mice with that in *LepR*-Cre; *Inhba*-floxed mice and *Csf1r*MeriCreMer; *Inhba*-floxed mice (Fig. 3f-h). Since preNeu-specific Cre mice are now unavailable, and our scRNA-seq analysis showed limited *Inhba* expression in preNeu at bone injury sites, we selected *Vav1*-Cre mice, in which Cre recombinase is expressed in HSCs to delete the *Inhba* gene in preNeu [24]. Vav1-Cre; Inhba-floxed mice were born in expected Mendelian ratio and grew normally. Bone induction at the injury sites was significantly impaired in the *Vav1*-Cre; *Inhba* fl/fl [herein called *Inhba* conditional knockout (*Inhba cKO*)] mice (Fig. 3f), but not in *LepR*-Cre; *Inhba* cKO (Fig. 3g) or *Csf1r*MerCreMer; *Inhba* cKO mice (Fig. 3h). These results were aligned with our reanalysis of scRNA-seq data, which showed that *Inhba* expression was not detected in osteoblast-lineage cells (Fig. S4a-c). qPCR analysis and reanalysis scRNA-seq data from osteoblastic-lineage cells revealed that activin A receptors, such as *Acvr1b* and *Acvr2a*, were expressed at higher levels in *LepR*(+) BMSCs than in *Bglap*-expressing mature osteoblasts (Fig. S4a, g, h). These findings suggests that preNeu-secreted activin A acts on LepR(+) BMSCs. This, in turn, promotes osteoblastic differentiation. The expression of a target gene of activin signaling Smad7 and an osteoblastic marker Sp7 were lower at bone injury sites 4dpi in Inhba cKO mice (Fig. 3i). The local application of activin A rescued the expression of Smad7 and Sp7 and bone induction in Clo-lip-treated mice (Fig. 3j, k). Thus, preNeu-derived activin A acts on *LepR*(+) cells, promoting bone fracture healing.

**Characterization of clo-lip-targeted F4/80(+)Csf1r(-) macrophages**

It has been reported that neutrophils are not depleted by Clo-lip treatment [25]. Then, we investigated which cell cluster is identical to F4/80(+)Csf1r(-) macrophages as the target of Clo-lip. The scRNA seq analysis showed that the Mac_1 cluster exhibited high levels of *Ccr2*,

*Ms4a4c* and *S100a4* (Fig. 4a), while the Mac_3 was identified by high expression of *Adgre4*, *S1pr5* and *Ace* (Fig. 4b). Pre. Ocl clusters highly expressed their classic marker genes (*Acp5*, *Tnfrsf11a* and *C3ar1*) (Fig. 4c). Among several macrophage subpopulations, Mac_1, became the largest Mφ subset after injury, increasing from 17.40% to 33.35%, whereas pre-osteoclasts and the adjacent Mac_3 subset remained unchanged (Fig. 4d). Cx3cr1 is predominantly expressed in monocyte/Mφs under anti-inflammatory rather than pro-inflammatory conditions but is not expressed in granulocytes [26-28]. We found that *Cx3cr1* was strongly expressed in Mac_1 and Mac_3 (Fig. 4e). Therefore, we divided F4/80(+) Csf1r(–) cell fraction into 3 populations based on Cx3cr1 expression to further identify a new subset of Mφs. Flow cytometric analysis of bone marrow cells from mice 3 days post-bone injury revealed an increased percentage of the F4/80(+) Cx3cr1$^{hi}$ Csf1r(–) (R3) fraction at the injury site compared to the uninjured site (Fig. 4f). Additionally, the R3 fraction was confirmed to be targeted by Clo-lip treatment (Fig. 4g).

We next investigated whether R3 fraction and Mac_1 cluster correspond to the same or largely overlapping cell population. We found that Mac_1 highly expressed *Ccr2* mRNA (Fig. 4h), a gene known to play a crucial role in Mφ migration during bone healing [29]. Therefore, we examined whether R3 cells also strongly express Ccr2 protein. Flow cytometry analysis revealed that Ccr2 expression was significantly upregulated in R3 cells from uninjured and injured sites, but not in R3 cells from intact bone marrow (c-uninjured) or F4/80(+) Csf1r(+) cells (Fig. 4i). These results suggest that F4/80(+) Cx3cr1$^{hi}$ Csf1r(–) R3 cells correspond to the Mac_1 cluster, and R3 cells may express Ccr2 in response to the injury stimuli, thereby promoting their accumulation at the injury site. Together, Mac_1 is nearly identical to F4/80(+) Csf1r(–) Cx3cr1$^{hi}$ Ccr2$^{hi}$ cells, a new kind of Mφs named "osteodirective Mφs" (OdMacs), played a crucial role in bone regeneration and induction. Next, we performed pseudotime analysis by using Monocle and predicted the differentiation of Mac_1. The analysis revealed that Mac_2 and 4 gave rise to Mac_1 as committed regenerative Mφs with high expression of *Cx3cr1* (Fig. 4j, k). As proliferating precursor cells, Mac_2 and Mac_4, progress toward the committed, non-proliferating Mac_1 population (Fig. 4l).

**The interaction between OdMac and preNeu promotes bone fracture healing**

Gene ontology (GO) enrichment analysis of Mac_1 revealed an accumulation of GO terms associated with chemoattractant activity and extracellular matrix structural components, highlighting the highly expression genes such as *Ccl9* and *Fn1* (Fig. 5a-c). These GO terms have been reported as crucial for optimal bone healing process [30, 31]. On the other hand, previously reported osteogenic factors such as BMP and Oncostatin M [32], were not expressed in Mφ lineage cells in our analysis (Fig. S3b). These findings suggest that OdMacs cooperate with activin A-expressing preNeu to promote bone regeneration.

To explore molecular interactions between Mac_1 and preNeu, we performed CellChat analysis based on our scRNA-seq data. This analysis revealed enrichment of Fn1 signaling, chemokines, and complement pathways between Mac_1 and preNeu (Fig. 5d), suggesting that Mac_1 may regulate preNeu (Pre_Neu1&2) migration through these pathways. Indeed, Clo-lip administration significantly reduced the number of preNeu at the injury site (Fig. 5e).

Activin A has been shown to induce periosteal mesenchymal cell proliferation [22]. In our study, Clo-lip suppressed *Inhba* mRNA expression and inhibited the proliferation of *LepR* Tom(+) cells at the injury sites (Fig. 2g, Fig S2a-c). Furthermore, reanalysis of scRNA-seq data during bone regeneration revealed increased expression of genes involved in Wnt signaling, such as *Wnt4*, *Lrp5*, and *Ctnnb1*, during osteoblastic differentiation. Notably, *Ctnnb1* was expressed even in *LepR*(+) reticular cells (BMSCs) (Fig. S4a). These findings prompted us to investigate whether Clo-lip suppresses Wnt/β-catenin signaling at the injury sites. qPCR showed that *Axin2* mRNA, a Wnt target gene, was decreased at injury sites in Clo-lip-treated mice compared with Ctrl-lip-treated mice at 4 dpi as well as in Vav1-Cre; Inhba cKO mice (Fig. 5f, g). In contrast, the local injection of recombinant activin A into the bone injury sites increased Axin2 expression in Clo-lip-treated mice (Fig 5h). Immunofluorescent analysis revealed that the percentage of β-catenin(+) *LepR* Tom(+) stromal cells were markedly reduced at the injury site in Clo-lip-treated mice (Fig. 5i). Immunostaining for Sp7 using *Axin2*-CreERT2; R26-Tomato mice [33, 34] showed that Clo-lip treatment significantly reduced the percentage of *Axin2*-Tom(+) Sp7(+) cells among all Sp7(+) cells at the injury site from 5 to 7 dpi (Fig. 5j). The injection of recombinant activin A restored the number of Wnt/β-catenin signaling(+) Sp7(+) cells, including BMSCs and osteoblasts at the injury sites of Clo-lip-treated mice (Fig. 5k, l). These data suggest that activin A induces the proliferation of *LepR* Tom(+) stromal cells, which in turn, promotes osteoblastogenesis by increasing Wnt/β-catenin signaling.

**Wnt4 secreted from BMSCs are essential for regenerative osteogenesis**

The findings from Axin2-Cre; tdTomato mice prompt us to investigate how Wnt/β-catenin signals are activated at the injury sites during bone regeneration and induction. qRT-PCR analysis showed that the expression of *Wnt4*, *5a*, *10b*, and *16* mRNA was higher at bone injury sites at 4 dpi (Fig. 6a). The expression of a Wnt antagonist, *Sost* mRNA was modestly lower at 4 dpi, but not those of Dickkopf family and secreted Frizzled-related protein family members (Fig. S7a). qRT-PCR analysis showed the expression of *Wnt4* mRNA was decreased at the injury sites of Clo-lip-treated mice, but *Wnt5a*, *Wnt10b*, *Wnt16* or *Sost* mRNAs were not (Fig. 6b, Fig. S7b). The expression of *Wnt4* mRNA peaked at 3 dpi during the early phase of the healing (Fig. 6c) coinciding with the peak in LepR(+) MSCs (Fig S2b), suggesting that stromal cell–derived Wnt4 is primarily involved in the expansion and differentiation of BMSCs. The local injection of activin A restored the expression of Wnt4 at 3 dpi in Clo-lip-treated mice (Fig. 6d). Furthermore, the overexpression of Wnt4, as well as a typical canonical Wnt3a increased an osteoblast marker, alkaline phosphatase activities in bone marrow stromal cell line ST2 cells and embryonic fibroblast line C3H10T1/2 (Fig 6e, Fig S6). Addition of Wnt/β-catenin signaling inhibitor sclerostin suppressed Wnt4-induced alkaline phosphatase activity (unpublished data of Y. Nakamichi), suggesting that Wnt4 is able to drive osteoblastic differentiation through Wnt/β-catenin signals.

Next, we investigated a source of Wnt ligands (especially Wnt4) during bone fracture healing. To this end, we analyzed bone injury sites in *LepR*-Cre; *Wls* cKO mice, in which

Wntless (Wls), an essential molecule for Wnt secretion [35] was specifically deleted in BMSCs. Compared to control mice, bone induction was dramatically impaired in the Wls cKO at 7 dpi (Fig. 6e). Furthermore, the number of LepR-Cre-tomato(+)Ki67(+) and LepR-Cre-tomato(+)*Col1*-GFP(+) cells were significantly reduced at the injury sites at 4 dpi, indicating that BMSC-derived Wnt ligands, probably Wnt4, are critical for both proliferation and osteogenic differentiation (Fig. 6f). Thus, LepR(+) BMSCs serve as a crucial source of Wnt ligands during the early stages of bone healing, and Wnt ligands promote differentiation of BMSCs into osteoblasts in an autocrine manner. OdMφs and PreNeu govern BMSC proliferation and differentiation through activin A-Wnt signaling in bone healing (Fig. 6h).

### Discussion

Mφs play a crucial role in tissue regeneration, including fracture healing, by interacting with various immune cells and tissue-resident stem cells [36, 37]. In this study, we identified a novel F4/80(+) Cx3cr1$^{hi}$ Csf1r(–) OdMacs specifically accumulating at bone injury sites. Depletion of OdMacs using Clo-lip impaired the bone fracture healing. We further demonstrated that communications among OdMacs, preNeu, and BMSCs facilitate bone fracture healing through localized activation of activin A-Wnt signaling axis. The depletion of OdMac disrupt the recruitment of preNeu, BMSCs, and newly bone formation at the bone injury sites. These findings highlight the critical role of OdMacs as key orchestrators of bone regeneration.

**Interaction between BMSCs and immune cells**

Previous studies [9] have shown that bone resident macrophages known as Osteomac interact with osteoblasts to support fracture healing and bone remodeling. However, a comprehensive understanding of the full spectrum of cell types involved in tissue remains incomplete. In this study, we identified PreNeu as additional key player in bone regeneration niche. The role of Neu in bone regeneration has been controversial [38, 39]. As reported in previous studies [23, 40], mature Neu [Ly6g(+) Ki67(–)] transiently appear at the early stages of bone injury and are markedly decreased by 3 dpi, suggesting their involvement primarily in the initial inflammatory response. Our immunofluorescent analysis and scRNA analyses, for the first time, reveled a critical role of activin A-expressing preNeu in the bone regeneration process. PreNeu [Ly6g(+) Ki67(+)] were increased at the injury site at 3 dpi in an OdMac-dependent manner and found to secrete activin A. Although Clo-lip reportedly do not deplete mature Neu, they can suppresse inflammatory functions of Neu, including Neu extracellular trap formation and inflammatory cytokine expression [25]. Therefore, we cannot fully exclude the possibility that Clo-lip affects Neu function by modulating their pro-inflammatory state during fracture healing. Further studies are warranted to delinate the potential anti-inflammatory effects of Clo-lip on fracture healings.

**Positive feedback loop between OdMacs and preNeu at bone injury sites**

The Cell Chat analysis revealed that OdMacs interact with PreNeu at bone injury sites through the Ccl9-CCR1 signaling axis. OdMac may recruit PreNeu to injury sites by establishing a Ccl9 concentration gradient. Ccr1, the receptor of Ccl9 play a crucial role in neutrophil recruitment during inflammatory conditions including arthritis. Notably, Ccr1 KO mice exhibit reduced arthritis severity [41], highlighting the importance of this signaling pathway. Moreover, Tgf-b signaling has been shown to induce Ccl9 expression in myeloid cells, likely mediated by Smad2/3 [42]. These findings suggest that PreNeu-derived activin A stimulates Ccl9 expression in OdMacs, which in turn promotes the accumulation of both OdMacs and PreNeus at the site of injury. Thus, a positive feedback loop is established between OdMac-derived Ccl9 and PreNeu-derived activin A, amplifying the recruitment of these cells during the second wave of response and facilitating bone fracture healing.

In our model, the first wave of Neus was disappeared within 3 days post-injury. Their role in bone fracture healing have been previously proposed. Neus have been shown to synthesize and deposit fibronectin into the extracellular matrix of the hematoma, forming a scaffold that facilitate the recruitment and migration of various cell types involved in fracture repair [43]. Additionally, fibronectin has also been reported to enhance the migration of human Neu [44, 45], suggesting that the fibronectin deposited during the initial Neu response may support the adhesion and migration of preNeu during the second wave of infiltration. Furthermore, human macrophages have been shown to express fibronectin [46, 47] indicating that OdMac may also contribute to fibronectin production at the injury site. This OdMac-derived fibronectin could facilitate the recruitment of preNeu and BMSCs, further supporting the cellular dynamics essential for effective bone fracture healing.

**Wnt signaling induces the differentiate of BMSCs in cell autonomous manner**

Wnt/β-catenin signaling plays a critical role in bone formation during skeletal development and bone remodeling [34, 35]. It also promotes the differentiation of Cxc12-positive BMSCs into osteoblasts during bone regeneration [48]. Bone fracture healing is impaired in BMSC-specific β-catenin cKO mice, indicating that activation of Wnt/β-catenin signaling in BMSCs is critical for their osteoblastic differentiation following bone injury. However, the source of Wnt ligands that activate this pathway has remained unclear. Our current study using Lepr-Cre; Wls-floxed mice clearly demonstrated that BMSC-derived Wnt ligands can drive the osteogenic differentiation of BMSC into osteoblasts in an autonomous manner. Notably, newly formed bone was localized around regions with accumulation of LepR-tdTomato -positive in the injured bone. This finding suggests two possibilities: (1) the proliferation of Wnt-secreting BMSCs may increase local Wnt ligand concentration, and (2) preNeu-derived factors, including activin A, may induce Wnt4 expression in BMSCs at the injury sites. This scenario is supported by previous finding showing that TGF-β signaling can induce Wnt4 expression in dermal fibroblasts [49]. Further investigations are needed to elucidate the mechanisms by which Wnt-expressing BMSCs are initially recruited or activated at the injury cites.

This study identified a novel regulatory role for OdMac in establishing a bone regenerative niche. OdMacs secrete both fibronectin and Ccl9, which together promote the recruitment of activin A-expressing preNeu to bone injury sites. This second wave of preNeus, in turn, enhances the proliferates and differentiation of BMSCs, thereby promoting bone regeneration. Thus, OdMacs are key cellular players in organizing a regenerative microenvironment and facilitating efficient fracture healing. The OdMac-regulated regenerative niche represents a promising therapeutic target for regenerative medicine. (4076 words/4300)

### Materials and methods

#### Experimental animals

*Col1(2.3)* -green fluorescent protein (GFP) mice [15] were kindly provided by Dr. Matsuo at Keio University. *LepR*-Cre [50], Rosa26tdTomato [51], *Csf1r*MerCreMer [52], *Csf1r*-flox [53], *Axin2*-CreERT2 [54], *Vav1*-Cre [55-57] and *Wls*-flox [58] mice were purchased from the Jackson Laboratory. The creation of the *Inhba*-flox mutant mice has been described [59], we appreciate the support of Drs. Takayanagi and Okamoto at Tokyo University. *Vav1*-Cre, *LepR*-Cre and *Csf1r*MerCreMer transgenic mice were bred with *Inhba* fl/fl mice to create homozygous *Inhba*-knockouts. All mice were maintained under specific pathogen-free conditions at 24 ± 2°C and 50% to 60% humidity with a 12-hours light/dark cycle, and were provided with sterilized water and ad libitum diets in animal facilities. All animal studies were approved by the Animal Management Committee of Matsumoto Dental University and animal experiments were performed with minimal suffering.

#### Mouse tibia intramembranous bone injury model

Mice aged 8 to 16 weeks were utilized for the study. Age- and sex-matched mice were used for all of the experiments unless otherwise noted. Following induction of general anesthesia, an incision was performed on the skin covering the tibia, exposing the cortical bone. Subsequently, a hole with a diameter of 0.8 mm was drilled into the cortical bone of the diaphysis using a 21-gauge needle. The needle was used to penetrate one side of the cortical bone, creating a bone marrow injury while keeping the other side intact. After the injury was induced, the wound was sutured. For local administration of recombinant activin A, the protein (PeproTech, 120-14E) was diluted in sterilized 0.1% BSA/PBS (100 ng/μl), and 10 μl was injected into the bone defect region at 3 dpi. The control group was injected with the same volume of BSA/PBS.

#### Micro-computed tomography (Micro-CT) analysis

Micro-CT analysis (ScanXmate-A080, Comscan Tecno, Kanagawa, Japan) was performed on mice to evaluate bone regeneration at the injury site at 7 or 8.5 dpi. Trabecular bone parameters at the injury site were quantified using image analysis software (TRI/3D-BON, Ratoc System Engineering, Tokyo, Japan).

**Depletion of Mφs during bone regeneration process**

The liposomes used in this study were obtained from Liposoma BV Co. Ltd. (Science Park 408, 1098XH AMSTERDAM, the Netherlands). Clodronate liposomes (Clo-lip) consist of artificially prepared lipid vesicles encapsulating clodronate, with a concentration of approximately 5 mg/ml in the suspension. Control liposomes (Ctrl-lip) consist of artificially prepared lipid vesicles encapsulating an aqueous PBS solution. All mice received intraperitoneal injections (10.0 μl/g) of Clo-lip or Ctrl-lip at the time of surgery, followed by administrations every two days until 1 or 2 days before tissue harvests.

AFS98 was purified from the ascites of mice inoculated with AFS98 hybridoma cells by affinity chromatography and administered to mice via an i.p. injection at a dose of 2 mg.

**Fluorescent DiI Liposomes administration**

Fluorescent DiI Liposomes (a suspension of artificially prepared lipid vesicles labeled with DiI encapsulating an aqueous PBS solution) was purchased from Liposoma BV and used for detecting phagocytes. Intraperitoneal injections (20.0 μl/g) of DiI were administered following the same protocol as Clo/Ctrl-lip administration.

**Induction of Cre-mediated recombination**

Tamoxifen (T-5648; Sigma) was dissolved in corn oil followed by sonication for 15 to 30 min at 37 ºC. The tamoxifen solution (about 2 mg of tamoxifen per 30 g of body weight) was intraperitoneally administered into *Axin2*-Cre ERT2; Rosa26 tdTomato mice for three consecutive days prior to tissue harvest, to *Csf1r*MerCreMer; *Csf1r* flox mice daily for one week, and *Csf1r*MerCreMer; *Inhba* flox mice once daily for 5 days, spanning from 2 days before injury to 2 days after injury.

**Histological analysis**

Samples were fixed in 4% paraformaldehyde (PFA) overnight at 4 ºC and then transferred into 20% sucrose in PBS for incubation at 4 ºC for more than 24 hours. Tissues were embedded in O.C.T. compound, and sections of 10 µm thickness were obtained using Kawamoto's film method with Cryofilm type ⅢC and a tungsten carbide knife (Section-Lab Co. Ltd. Hiroshima, Japan). The sections were incubated with primary antibodies overnight at 4 ºC and then with secondary antibodies at room temperature for 2 hours. Finally, the sections were mounted using 30% glycerol, covered with coverslips, and sealed with nail polish. Fluorescence images were acquired using a confocal laser scanning system (LSM 510; Carl Zeiss, Oberkochen, Germany).

Fluorescent staining of osteoclasts was performed on frozen sections using the ELF 97 Endogenous Phosphatase Detection Kit (Invitrogen) in combination with TRAP buffer.

**Antibodies and other reagents**

Primary antibodies included anti-F4/80 rat monoclonal (BM8, sc-52664, Santa Cruz Biotechnology 1:25), anti-F4/80 rabbit monoclonal (D4C8V, 30325s, Cell Signaling, 1:400), anti-F4/80 FITC rat monoclonal (CI: A3-1, ab105155, Abcam, 1:100), anti-CD115 sheep polyclonal (AF3818, R&D Systems, 1:100), anti-CD115 APC rat monoclonal (AFS98, eBioscience, 1:100), anti-CD115 PE rat monoclonal (AFS98, eBioscience, 1:100), anti Ki-67 FITC rat monoclonal (SolA15, eBioscience, 1:100), anti-Cx3cr1 PeCy7 mouse monoclonal (149015, BioLegend, 1:100), anti-Ccr2 PE rat monoclonal (150609, BioLegend, 1:100), anti-Ly-6G FITC rat monoclonal (1A8, 88876s, Cell Signaling, 1:100), anti-Cxcr2 APC rat monoclonal (149613, BioLegend, 1:100), anti-beta Catenin rabbit monoclonal (E247, ab32572, Abcam, 1:100), anti-Sp7 rabbit polyclonal (ab22552, Abcam, 1:200), purified rat IgG2b monoclonal (400601, BioLegend), purified normal rabbit IgG (148-09551, FUJIFILM) and purified sheep IgG (5-001-A, R&D Systems) antibodies. As secondary antibodies, we employed FITC-conjugated donkey anti-rat IgG (AP189F, 596 Sigma-Aldrich, 1:200), Alexa Fluor® 488-conjugated donkey anti-rabbit IgG (ab150073, Abcam,1:500), Alexa Fluor® 488-conjugated donkey anti-sheep IgG (ab150177, Abcam, 1:500), Alexa Fluor® 594- conjugated donkey anti-rabbit IgG (A21207,

Invitrogen, 1:500), and Alexa Fluor® 594-conjugated donkey anti-rat IgG (A21209, Invitrogen 1:500). Can Get Signal (NKB-601, TOYOBO) was used to enhance immunoreactions. Nuclei were counterstained with TO-PRO-3 Iodide (642/661, T3605, Invitrogen) or Propidium Iodide Solution (421301, BioLegend).

**Flow cytometry and cell sorting**

Preparation and staining of bone marrow hematopoietic cell suspension: bone marrow cells were flushed out from mouse tibia using a 1 mL syringe filled with cell culture medium (α-MEM supplemented with 10% FBS and L-Glutamine). The cell suspension was then centrifuged at 5000 rpm for 5 minutes. After centrifugation, the supernatant was removed, and the pellet was resuspended in 1 X RBS lysis buffer (eBioscience) and incubated on ice for 3 minutes. An equal volume of culture medium was added to neutralize the lysis buffer, followed by centrifugation. The supernatant was discarded, and the pellet was washed twice with culture medium by repeating the centrifugation steps to ensure complete removal of the lysis buffer. After the final wash, the cell pellet was resuspended in diluted fluorescence-conjugated primary antibody solution and incubated on ice for 30 minutes in the dark. Following incubation, the cells were washed again by centrifugation, the supernatant was removed, and the pellet was resuspended in culture medium. The stained cell suspension was stored on ice in the dark and promptly subjected to flow cytometry analysis. Dead cells and debris were excluded by FSC, SSC and SYTOX red dead cell (Thermo Fisher Scientific) staining profiles. Flow cytometry and cell sorting were performed using Cytomics FC500; Beckman Coulter, Fullerton, CA FACSMelody (BD Bioscience) and FACSAriaIlu cell sorter. Data were analyzed with FlowJo software.

**RNA isolation and quantitative real-time PCR.**

Samples of bone tissue from the bone injury site of mouse tibiae and control samples from the uninjured region of the contralateral tibiae were homogenized in TRIzol Reagent (Thermo Fisher Scientific, Waltham, MA, USA) using the TissueLyser II (Qiagen, Hilden, Germany). Total RNA was purified using RNA isolation kits (NucleoSpin RNA, Macherey-Nagel,

Düren, Germany). cDNA was synthesized from purified RNA with the iScript gDNA Clear cDNA Synthesis Kit (BIO-RAD) according to the standard protocol. Real-time RT-PCR was performed using Fast SYBR Green (Thermo Fisher Scientific) and the StepOnePlus system (Thermo Fisher Scientific). The PCR conditions were as follows: 95 ºC for 20 s for denature, followed by 40 cycles of 95 ºC for 3 s and 60 ºC for 30 s. Gene expression data were normalized to Gapdh. The sequences of the primers (mouse) for each gene were as follows:

*Gapdh*, 5′-TGTGTCCGTCGTGGATCTGA-3′ (forward) and
5′-TTGCTGTTGAAGTCGCAGGAG-3′ (reverse);

*Sp7*, 5′- CGCATCTGAAAGCCCACTTG-3′ (forward) and
5′-CAGCTCGTCAGAGCGAGTGAA′ (reverse);

*Inhba*, 5′- GGAGAACGGGTATGTGGAGA-3′ (forward) and
5′-TGGTCCTGGTTCTGTTAGCC′ (reverse);

*Axin2*, 5′- ATGTCCTGTCTGCCAGCGTTC-3′ (forward) and 5′-
CAAGCACTAGCCAGTGGGTCAA-3′ (reverse);

*Smad7*, 5′- GTGTTGCTGTGAATCTTACGG-3′ (forward) and
5′-GATGAAGATGGGGTAACTGCT-3′ (reverse);

*Acvr2a*, 5′- GTTACACCGAAGCCACCCTA-3′ (forward) and
5′-AACCAAATCTTCCCCTTGCT-3′ (reverse);

*Acvr2b*, 5′- TTAAGGATCACTGGCTGAAACA-3′ (forward) and
5′-GGATACCCGCTCTTCTACACAG-3′ (reverse).

*Wnt1*, 5′- ATAGCCTCCTCCACGAACCT-3′ (forward) and
5′-GGAATTGCCATTTGCACTCT-3′ (reverse);

*Wnt2*, 5′- GGTCAGCTCTTCATGGTGGT-3′ (forward) and
5′-ATCTCTGTCCAGGGTGTTGC-3′ (reverse);
*Wnt2b*, 5′- TCAACGCTACCCAGACATCA-3′ (forward) and
5′-ACCACTCCTGCTGACGAGAT-3′ (reverse);
*Wnt3*, 5′- AGGAGTGCCAGCATCAGTTC-3′ (forward) and
5′-ACTTCCAGCCTTCTCCAGGT-3′ (reverse);
*Wnt3a*, 5′- CTGGCAGCTGTGAAGTGAAG-3′ (forward) and
5′-TGGGTGAGGCCTCGTAGTAG-3′ (reverse);
*Wnt4*, 5′- CTGGAGAAGTGTGGCTGTGA-3′ (forward) and
5′-CAGCCTCGTTGTTGTGAAGA-3′ (reverse);
*Wnt5a*, 5′- CAAATAGGCAGCCGAGAGAC-3′ (forward) and
5′-CTCTAGCGTCCACGAACTCC-3′ (reverse);
*Wnt5b*, 5′- CTGCTTGCGTAATGAGACCA-3′ (forward) and
5′-AAAGCAACACCAGTGGAACC-3′ (reverse);
*Wnt6*, 5′- TCAGTTCCAGTTCCGTTTCC-3′ (forward) and
5′-CATGGAACAGGCTTGAGTGA-3′ (reverse);
*Wnt7a*, 5′- GGTGCGAGCATCATCTGTAA-3′ (forward) and
5′-TCCTTCCCGAAGACAGTACG-3′ (reverse);
*Wnt7b*, 5′- AAGCCTATGGAGACGGACCT-3′ (forward) and
5′-TTGGTGTACTGGTGCGTGTT-3′ (reverse);
*Wnt8a*, 5′- AGCACAGAGGCTGAGCTGAT-3′ (forward) and

5′-TCTGCTCTCCTCTCCTCCAC-3′ (reverse);

*Wnt8b*, 5′- GTGGACTTCGAAGCGCTAAC-3′ (forward) and
5′-CTGCTTGGAAATTGCCTCTC-3′ (reverse);

*Wnt9a*, 5′- TGCTTTCCTCTACGCCATCT-3′ (forward) and
5′-CCTTGACAAACTTGCTGCTG-3′ (reverse);

*Wnt9b*, 5′- TGGAGCGCTGTACTTGTGAC-3′ (forward) and
5′-GCACTTGCAGGTTGTTCTCA-3′ (reverse);

*Wnt10a*, 5′- CATGAGTGCCAGCATCAGTT-3′ (forward) and
5′-ACCGCAAGCCTTCAGTTTAC-3′ (reverse);

*Wnt10b*, 5′- GGAAGGGTAGTGGTGAGCAA-3′ (forward) and
5′-CTCTCCGAAGTCCATGTCGT-3′ (reverse);

*Wnt11*, 5′- CAGGATCCCAAGCCAATAAA-3′ (forward) and
5′-GTAGCGGGTCTTGAGGTCAG-3′ (reverse);

*Wnt16*, 5′- TCCTGCTAGCCATGAATCTACACAA-3′ (forward) and
5′-AGCGGCAGTCCACAGACATTAAC-3′ (reverse);

*Dkk1*, 5′- CAGGTGTGCACCAAGCACAA-3′ (forward) and
5′-TCTGGCAGGTGTGGAGCCTA-3′ (reverse);

*Dkk2*, 5′- AATACAAGCACAGGGCAGAATTGTC-3′ (forward) and
5′-GTATGTGCTCATCCCGTCATTCAG-3′ (reverse);

*Dkk3*, 5′- CAAAGTCGCTTAGCAACAATGGAA-3′ (forward) and
5′-TGGCACCTGAAACCGTCATC-3′ (reverse);

*Dkk4*, 5′- ACACAAGGCCAGTGATGGACAG-3′ (forward) and

5′-GCGAGCACAGCAAAGTCCAG-3′ (reverse);

*Sfrp1*, 5′- TGACCAGCGTTGCCAATGA-3′ (forward) and

5′-CACCAACCATGAGCTCTGAGGA-3′ (reverse);

*Sfrp2*, 5′- AGGACGACAACGACATCATGG-3′ (forward) and

5′-GTTCAGCTTGTAAATGGTCTTGTTC-3′ (reverse);

*Frzb* (*Sfrp3*), 5′- CCGACACCTTGGACTGGGTAA-3′ (forward) and

5′-GGTAAACAATCGGTCACGAGCA-3′ (reverse);

*Sfrp4*, 5′- AAGCCGACCCTGGCAACATA-3′ (forward) and

5′-TTGTGACCTCATTGCAACCACTC-3′ (reverse);

*Sfrp5*, 5′- GCCCAGTGTGAGATGGAGCA-3′ (forward) and

5′-CTTCTGGGCTCCAATCAACTTTC-3′ (reverse);

*Sost*, 5′- TCCTGAGAAGAACCAGACCA-3′ (forward) and

5′-GCAGCTGTACTCGGACACATC-3′ (reverse);

**RNA-Seq and analysis**

The data analysis was performed by Genble Inc., Fukuoka, Japan. Samples (S1-S3) were isolated RNA from sorted F4/80(+) Csf1r(+) bone marrow cell fraction, while samples (S4-S6) were isolated RNA from sorted F4/80(+) Csf1r(-) bone marrow cell fraction. All RNA samples (S1–S6) passed the quality control check, ensuring they meet the required standards for sequencing. Library preparation and sequencing: for library preparation, the SMART-Seq v4 Ultra Low Input RNA for Sequencing kit was used. Sequencing was performed on the Illumina NovaSeq 6000 platform, generating paired-end reads with a read length of 100 base pairs (bp).

Base calling was conducted using the Illumina Real-Time Analysis (RTA) software, and demultiplexing was carried out using the Illumina bcl2fastq tool. Data cleaning: raw sequencing reads underwent multiple preprocessing steps to ensure data quality. Poly A/T sequences (≥5 consecutive A/T bases at the 3' end) were removed, along with adapter sequences from the sequencing library preparation kit. Quality filtering was applied by trimming low-quality bases, removing bases with a quality score below Q20 at the read ends, and filtering out four-base windows with an average quality below Q15. Additionally, short reads (<36 bases) were excluded from further analysis. Reference transcriptome and annotation: gene expression was quantified using the Gencode GRCm39vM27 reference transcriptome. Gene annotations were obtained from the Ensembl database (Ensembl Genes version 106) to ensure accurate mapping and expression profiling. Gene expression quantification and normalization: gene and transcript expression levels were estimated and normalized using the Transcripts Per Million (TPM) method. TPM normalization accounts for transcript length and sequencing depth, enabling more accurate comparisons of gene expression across samples. Differential expression analysis: differential gene expression analysis was performed using edgeR (version 3.26.8). Genes with TPM ≤ 1 across all samples were excluded from analysis. Normalization was conducted using the Trimmed Mean of M-values (TMM) method. Statistical significance was assessed using the exact test, and multiple testing correction was applied using the Benjamini & Hochberg (BH) method to control the false discovery rate. Differentially expressed genes (DEGs) were identified based on an adjusted p-value < 0.05 and an absolute log2 fold change (|log2FC|) ≥ 0.5. Computational Tools: the following computational tools were used for quality control, read processing, expression quantification, and differential expression analysis: FastQC, PRINSEQ++, Trimmomatic, Salmon, tximport and EdgeR. Heatmap data is displayed by using Heatmapper [60].

**ScRNA-Seq and analysis**

The data of "bacorde feature matrix" file was generated by Genble Inc., Fukuoka, Japan and further downstream analysis steps were performed by the authors.

F4/80(+) and Csf1r(+) cells were sorted using FACSMelody from both the bone injury site (3 dpi) and the uninjured site of the injured tibia. Each sample (injured/uninjured) was pooled from cells isolated from six tibiae of three mice. The experiment included both male and female subjects, acknowledging the potential for gender differences. All cell samples passed the quality check performed by Genble. Library Preparation and Sequencing: single-cell isolation, library preparation, and sequencing were performed according to the following conditions. Single-cell suspensions were generated using the 10x Genomics Chromium System, followed by library preparation with the Chromium Single Cell 3' Library & Gel Bead Kit. Library preparation was conducted according to the standard protocol provided by 10x Genomics. The quality of the prepared libraries was assessed using BioAnalyzer 2100 (Agilent) and LabChip (PerkinElmer). Sequencing was performed on the DNBSEQ-G400 (MGI) platform. Demultiplexing, Mapping, and Annotation: raw sequencing reads were demultiplexed, and barcode mapping was performed under the following conditions. Reads were aligned to the custom Gencode GRCm39 reference genome using the Cell Ranger (v6.1.2) pipeline. Gene annotation was based on the custom Gencode vM31 annotation dataset.

Further downstream computational analysis steps were performed using the Seurat (version 5.1.0) and Monocle (version: 2.32.0) R package. Genes expressed in less than five cells and cells expressing fewer than 200 genes were removed by primary filtering. After quality control, cells expressing more than 15% mitochondrial were excluded. The downstream analysis steps include read counts normalization, scale, dimensionality reduction and clustering using the packages of harmony (version 1.2.1) and clustree (version 0.5.1). The Uniform Manifold Approximation and Projection dimensional reduction technique was applied to visualize the clusters at a resolution of 0.4. The cell–cell communication was analyzed by using the CellChat (version 1.6.1). Clusters of Mono / Mφs were isolated for cell trajectory analysis.

The GSE136973 dataset was reanalyzed using previously described methods (Matsushita et al., 2020). The updated R package Monocle3 was utilized for this analysis.

**Gene set enrichment analysis**

The top 100 genes in Mac-1, ranked by p-value, were used for GSEA analysis with Metascape [61] and Enrichr.

**Cell culture, transfection, and alkaline phosphatase (ALP) staining**

ST2 and C3H10T1/2 cell lines were obtained from RIKEN BRC (Tsukuba, Japan) and ATCC (Manassas, VA, USA), respectively. Both cell lines were cultured in MEMα supplemented with 10% fetal bovine serum (FBS) and 1× Antibiotic-Antimycotic (Sigma) at 37°C in a 5% $CO_2$ atmosphere. Cells were seeded in 96-well plates and transfected with expression vectors: pCMV6-mWnt3a (Origene, #MC209652) and pCMV6-mWnt4 (Origene, #MC209653). Twenty-four hours post-transfection, the medium was replaced with differentiation medium consisting of MEMα supplemented with 10% FBS, 1× Antibiotic-Antimycotic, 10 μg/mL ascorbic acid, and 0.5 mM β-glycerophosphate. ST2 and C3H10T1/2 cells were cultured for an additional four and seven days respectively under standard conditions to induce osteoblast differentiation. Cells were then fixed with 4% PFA and permeabilized using 0.1% Triton X-100. ALP staining was performed using the BCIP/NBT substrate kit (Vector Laboratories) according to the manufacturer's instructions. pEGFP-N1 (Clontech) were used as control vectors where applicable.

**Statistical analysis**

In the experiments reported in this study, a minimum of three independently treated animals were used per condition. Data were evaluated using unpaired Student’ s t-tests. Fluorescence images were analyzed with ZEN3.2 (blue and black editions). Statistical analyses were performed with GraphPad Prism 9 (GraphPad Software Inc., La Jolla, CA). A P-value less than 0.05 was considered to be significant.

**Artificial Intelligence**

We used ChatGPT to correct grammatical errors, typos on the manuscript.

**Acknowledgments**

*Col1(2.3)* - GFP mice were kindly provided by Dr. Koichi Matsuo (Keio University). This work was supported by Grants-in-Aid 23K19731(Z.H.), 24K19848 (Z.H.), 24H00653 (Y.K.), 22K18402 (Y.K.), and 24K02629 (N.U.) from the Ministry of Education, Culture, Sports Science, and Technology of Japan and by a grant from Naito foundation and from Terumo life Science foundation (Y.K.). The *Inhba*-floxed mutant mice were created with the support of grant (R01HD032067 to M.M.M.) from the Eunice Kennedy Shriver National Institute of Child Health and Human Development.

**Author contributions**

Y.K. initiated and supervised the project. Y.K. wrote and reviewed the manuscript. Z.H. provided the study concept and design, performed experiments, collected data, maintained experimental animals, and wrote the manuscript.  L.S., K.M, T.M, S.U., Z.T., T.H. and N. U supported to maintain experimental animals and contributed to the data analysis. Y.M. and H.M. reanalyzed the scRNA-seq database. Y.N. performed cell cultures and transfection experiments. S.I, R.I, T.M, R.L., and M.K. contributed to cell sorting experiments and experimental animal maintenance. Y.N, K.M, K.O, H.T., and M.M. provided the transgenic mice and contributed to the data analysis.

**Competing interests**

The authors declare no competing interests.

**Figure. 1 Clodronate liposome administration, but not AFS98 or the macrophage-specific deletion of the *Csf1r* gene impairs bone healing.**

**(a)** Experimental protocol for clodronate liposome (Clo-lip) and control liposome (Ctrl-lip) injections.

**(b)** Immunofluorescent analysis of F4/80(+) cells at the bone injury site at 7 dpi in Clo-lip and Ctrl-lip-treated mice (n = 3 mice per each group).

**(c)** Micro-CT analysis of the bone injury site on 7 dpi during bone regeneration in mice treated with AFS98 and control antibodies (n = 3 mice per each group).

**(d)** Experimental protocol for AFS98 and saline injections.

**(e)** Immunofluorescent analysis of F4/80(+) cells at the bone injury site at 7 dpi (n = 3 mice per each group).

**(f)** Micro-CT analysis of the bone injury site on 7 dpi during bone regeneration, n = 3 per each group.

**(g)** Experimental protocol for *Csf1r* conditional knockout mice. Experimental group included *Csf1r*MerCreMer; *Csf1r* fl/fl (cKO) mice and control mice (*Csf1r*MerCreMer; *Csf1r* +/+ / fl/+ or *Csf1r*MerCreMer (–); *Csf1r* fl/+).

**(h)** Micro-CT analysis of the bone injury site on 7 dpi during bone regeneration (n = 5 mice per each group).

Scale bars; 100 µm in (**b**) and (**e**), 600 µm in (**c**) and (**f**). Parameters including BV/TV, Tb.Th, Tb. N, Tb. Sp were assessed in the injured region in **c, f,** and **h**. Data are shown in mean± S.D.. Statistical significance was determined by unpaired Student's t-tests. P- values are shown in each graph.

**Figure. 2 F4/80(+)Csf1r(-) phagocytic cell fraction is target for clodronate liposomes and facilitate bone healing.**

**(a)** Flow cytometry analysis of Mφ subpopulations from Clo-lip and AFS98-treated mice (n = 3 mice per each group).

**(b)** Flow cytometry analysis of F4/80 or Csf1r expression in DiI (+) cells.

**(c)** Immunofluorescent analysis of the percentage of DiI (+) signals in F4/80(+) cells or Csf1r(+) cells (white arrow heads) at the bone injury site at 4 dpi (n = 3 mice per group).

**(d)** Immunofluorescent analysis of F4/80(+)Csf1r(+) and F4/80(+)Csf1r(–) cells at the bone injury site and uninjured region at 4 dpi. White arrow heads indicate F4/80(+)Csf1r(+) cells. Yellow arrow heads indicate F4/80(+)Csf1r(–) cells (n = 3 mice per each group).

**(e)** RNA sequence analysis of F4/80(+)Csf1r(+) and F4/80(+)Csf1r(–) cell fractions. Heatmaps display the top 20 upregulated genes encoding secreted protein.

**(f)** Relative expression (TPM: transcripts per million) of *Inhba*, *Inhbb* and *Inha* in F4/80(+)Csf1r(+) and F4/80(+)Csf1r(–) cell fraction (n = 3 mice per each group).

**(g)** Quantitative RT-PCR analyses of *Inhba* mRNA expression from bone tissues from bone injury site (4 dpi) of mouse tibiae treated with Ctrl-lip or Clo-lip, and from the uninjured contralateral tibiae of Ctrl-lip and Clo-lip-treated mice (n = 5 mice per each group).

**(h)** Forward scatter (FSC) and side scatter (SSC) analysis of F4/80(+)Csf1r(+), F4/80(+)Csf1r(–), and F4/80(+) cell fractions.

Scale bars, 100 µm in (**c**) and (**d**). Data are shown in mean± S.D. Statistical significance was determined by unpaired Student's t-tests. P- values are shown in each graph (****: $p < 0.0001$).

**Figure. 3 PreNeu are the main source of activin A to promote bone repair.**

**(a)** Uniform manifold approximation (UMAP) visualization of 16 clusters of the isolated cells from the bone injury site (3 dpi) of mouse tibiae and uninjured site within the same injured tibiae.

**(b)** Expression pattern of *Inhba*, *Ly6g* and *Cxcr2* in the UMAP visualization. The dashed line enclosed the PreNeu

**(c, d)** Immunofluorescent analysis of Ly6g(+)Ki67(+) and Ly6g(+)Ki67(–) cells at the bone injury site, boundary region, and uninjured site at 1 **(a)** and 3 dpi **(b)**. Dash lines indicate the boundary between injury site and uninjured bone marrow. Yellow arrow heads indicate Ly6g(+)Ki67(+) cells.

**(e)** Flow cytometry analysis of Ly6g(+)Cxcr2(–) cells (preNeu) from uninjured and injured sites at 3 dpi.

**(f-h)** Micro-CT analysis of the bone injury site at 7 dpi in **(m)** *Vav1*-Cre; *Inhba* fl/fl (cKO) mice and *Vav1*-Cre; *Inhba* +/+ or *Vav1*-Cre (–); *Inhba* fl/+ / fl/fl mice (Ctrl) mice, , n = 8 per each group**,** in *LepR*-Cre; *Inhba* fl/fl (cKO) mice and *LepR*-Cre; *Inhba* +/+ or **(n)** *LepR*-Cre (–); *Inhba* fl/+ / fl/fl mice (Ctrl) mice, n = 7 per each group, and **(o)** *Csf1r*MerCreMer; *Inhba* fl/fl (cKO) mice and *Csf1r*MerCreMer; *Inhba* +/+ or *Csf1r*MerCreMer (–); *Inhba* fl/+ / fl/fl mice (Ctrl) mice n = 5 per each group.

**(j)** Quantitative RT-PCR analyses of *Smad7* and *Sp7* mRNA expression from bone injury sites of *Vav1*-Cre; *Inhba* fl/fl (cKO) mice and *Vav1*-Cre; *Inhba* +/+ or *Vav1*-Cre (–); *Inhba* fl/+ / fl/fl mice (Ctrl) mice, , n = 6 per each group.

**(j)** Quantitative RT-PCR analyses of *Smad7* and *Sp7* mRNA expression from bone injury sites treated with Ctrl and Clo-lip at 3 dpi, 6 hours after injections of recombinant activin A and vehicles (n = 4 mice per each group).

**(k)** Bone volume/ tissue volume (BV/TV) at the bone injury site at 8.5 dpi from Clo-lip-treated mice with or without the local injection of recombinant activin A (n = 6 mice per each group). Scale bars with its value are shown in each image. Data are shown in mean+ S.D. Statistical significance was determined by unpaired Student's t-tests.

**Figure. 4 Identification of bone regenerative F4/80(+)Csf1r(-)Cx3cr1$^{hi}$Ccr2$^{hi}$ Mφs through single cell RNA-Seq analysis.**

**(a)** Expression patterns of Mac_1 marker gene (*Ccr2, Ms4a4c* and *S100a4*).

**(b)** Expression patterns of Mac_3 marker gene (*Adgre4*, *S1pr5* and *Ace*).

**(c)** Expression patterns of Pre. Ocl marker gene (*Acp5*) and Mφs marker gene (*Cx3cr1*) in the UMAP visualization.

**(d)** Frequency of Mac_1 and Mac_3 plus Pre. Ocl clusters in uninjured and injured sites.

**(e)** Expression patterns of Mφs marker gene (*Cx3cr1*) in the UMAP visualization.

**(f)** Flow cytometry analysis of Cx3cr1 expression in F4/80(+)Csf1r(–) cell fraction isolated from bone marrow with or without bone injury (3 dpi). R1: Cx3cr1$^{low}$, R2: Cx3cr1$^{Int}$, R3: Cx3cr1$^{high}$. C-uninjured: bone marrow cells from contralateral tibiae, uninjured: cells from uninjured region of the injured mouse tibia, 3 dpi: cells from injured region of the injured mouse tibia at 3 dpi (n = 7 mice per each group).

**(g)** Flow cytometry analysis of R3 cells from injury sites treated with Ctrl-lip and Clo-lip (n = 6 mice per each group).

**(h)** Expression patterns of Mac_1 marker gene (*Ccr2*) in the Violin plot.

**(i)** Flow cytometry analysis of Ccr2 expression in Mφ-lineage cell subsets (all F4/80(+)) in c-uninjured region, uninjured sites, and injured sites at 3 dpi (n = 6 mice per each group), MFI: Mean fluorescence intensity.

**(j and k)** Pseudotime and single cell trajectory analysis by Monocle.

**(l)** Expression pattern of *Mki67* in the UMAP visualization. The dashed line enclosed the Mac_1 (OdMac). The arrow indicates the predicted cell differentiation trajectory.

Data are shown in mean± S.D. Statistical significance was determined by unpaired Student's t-tests. P- values are shown in each graph.

**Figure. 5 The interaction between OdMac and preneutrophils promotes bone healing via activin A-Wnt signaling axis.**

**(a)** Top 9 GO terms that were enriched in the cluster of Mac_1 cluster, analyzed using Metascape.

**(b)** Expression patterns of *Ccl9* a representative chemoattractant gene in the UMAP visualization.

**(c)** Expression patterns of *Fn1* a representative extracellular matrix structural constituent gene, in the UMAP visualization. The dashed line encloses the clusters of Mac_1 clusters in **(b)** and **(c)**.

**(d)** Cellchat analysis of signaling interactions from the Mac_1 cluster to the Pre. Neu_1 and Pre. Neu_ 2 clusters.

**(e)** Flow cytometry analysis of the absolute number of preneutrophils in Clo-lip and Ctrl-lip-treated mice (n = 4 mice per each group).

**(f)** Quantitative RT-PCR analyses of *Axin2* mRNA expression in bone tissues from bone injury sites (4 dpi) and uninjured region of contralateral tibiae (ctrl at 4dpi) treated with Ctrl-lip and Clo-lip (n = 4 mice per each group).

**(g)** Quantitative RT-PCR analyses of *Axin2* mRNA expression from bone injury sites of *Vav1*-Cre; *Inhba* fl/fl (cKO) mice and *Vav1*-Cre; *Inhba* +/+ or *Vav1*-Cre (–); *Inhba* fl/+ / fl/fl mice (Ctrl) mice, , n = 6 per each group.

**(h)** Quantitative RT-PCR analyses of *Axin2* mRNA expression from bone injury sites treated with Ctrl and Clo-lip at 3 dpi, 6 hours after injections of recombinant activin A and vehicles (n = 4 mice per each group).

**(i)** Immunofluorescent analysis of β-catenin(+) cells in *LepR*-Cre; tdTomato mice at bone injury sites at 4 dpi treated with Ctrl-lip and Clo-lip. Quantification of the number of β-catenin(+) *LepR*-Cre-labeled Tomato(+) cells (left) and the percentage of β-catenin(+)*LepR*-Cre-labeled Tomato(+) cells among all Tomato(+) cells (right) (n = 3 mice per each group).

**(j)** Immunofluorescent analysis of Sp7(+) *Axin2*-Cre-labeled Tomato(+) cells at the bone injury site at 5, 6, and 7 dpi treated with Ctrl-lip and Clo-lip. Quantification of the number of *Axin2*-Cre-labeled Tomato(+) cells (upper right) and the percentage of Sp7(+) Tomato(+) cells among all Sp7(+) cells (lower right) from 5 to 7 dpi (n = 3 mice per each group).

**(j, k)** Quantification of *Axin2*-Cre-labeled Tomato(+) cells (left) and the percentage of Sp7(+) Tomato(+) cells among all Sp7(+) cells (right) at bone injury sites at 5 dpi treated with ctrl-lip, Clo-lip, and Clo-lip plus recombinant activin A (n = 3-4 mice per each group). Scale bars with its value are shown in each image. Data are shown in mean$\pm$ S.D. Statistical significance was determined by unpaired Student's t-tests. P- values are shown in each graph (*; p < 0.05; **: p < 0.01).

**Figure. 6 Wnt4 produced by BMSCs contribute to regenerative osteogenesis in inflammatory phase.**

**(a)** Quantitative RT-PCR analyses of all 19 Wnts ligands mRNA expression in bone tissues from bone injury sites (4 dpi) and uninjured region of contralateral tibiae (ctrl at 4dpi). n = 3 mice per each group.

**(b)** Quantitative RT-PCR analyses of *Wnt4*, *5a*, *10b* and *16* mRNA expression in bone tissues from bone injury sites (4 dpi) and uninjured region of contralateral tibiae (ctrl at 4dpi) treated with Ctrl-lip and Clo-lip (n = 4 mice per each group).

**(c)** Quantitative RT-PCR analyses of *Wnt4* mRNA expression in bone tissues from bone injury sites at 0, 2, 3, 4 and 6 dpi, (n = 3 - 4 mice per each group).

**(d)** Quantitative RT-PCR analyses of *Wnt4* mRNA expression from bone injury sites treated with Ctrl and Clo-lip at 3 dpi, 6 hours after injections of recombinant activin A and vehicles (n = 4 mice per each group).

**(e)** Detection of ALP activity of ST2 cells after transfection by empty vector, mWnt4 and mWnt3a for 24 hours, and cultured in 96-well-plate for 4 days.

**(f)** Micro-CT analysis of the bone injury site on 7 dpi during bone regeneration in *LepR*-Cre; *Wls* fl/fl (cKO) mice and control mice (*LepR*-Cre; *Wls* +/+ / fl/+ or *LepR*-Cre (–); *Wls* fl/+), n = 5-6 mice per each group. Parameters including BV/TV, Tb.Th, Tb. N, Tb. Sp were assessed in the injured region. Scale bars; 600 µm.

**(g)** Immunofluorescent analysis of Ki67(+) cells in *LepR*-Cre; *Wls* fl/+ (Ctrl) or *Wls* fl/fl (cKO) tdTomato; *Col1*-GFP mice at the bone injury site and uninjured site at 4 dpi. Dash lines indicate the cortical bone surface. Scale bars with its value are shown in each image. Data are shown in mean+ S.D. Statistical significance was determined by unpaired Student's t-tests. P- values are shown in each graph (*; $p < 0.05$; ***: $p < 0.005$).

**(h)** Schematic diagram of the OdMac subset in early bone repair**.** F4/80(+) Csf1r(-) Cx3cr1hi Ccr2hi, which we term osteodirective macrophages (OdMac). OdMac are highly abundant in the early stages of bone injury sites and appear to be indispensable for regenerative bone induction. Mechanistically, OdMac accumulate activin A-expressing preneutrophils to the site of bone injury. This interaction enhances the proliferation and differentiation of LepR(+) bone marrow stromal cells (BMSCs) and activates Wnt signaling, thereby promoting bone regeneration.

**Figure. S1 Mφ subpopulations distinguished by F4/80 and Csf1r expression, and osteoclasts targeted by various depletion strategies.**

**(a)** Immunofluorescent analysis of *Col1*-GFP(+) cells at the bone injury site at 7 dpi, n = 3.

**(b)** Immunofluorescent analysis of osteocalcin(+) cells at the bone injury site at 7 dpi, n = 3.

**(c- f)** Immunofluorescent analysis of F4/80(+)Csf1r(+) and F4/80(+)Csf1r(–) cells at the bone injury sites at 7 dpi from Ctrl-lip or Clo-lip-treated (**c, e**), and AFS98 or saline-treated mice (**d, f**), n = 3.

**(g- j)** Immunofluorescent analysis of F4/80(+)Csf1r(+) and F4/80(+)Csf1r(–) cells at the bone injury site at 4 and 7 dpi in *Csf1r*MerCreMer; *Csf1r* fl/fl and control mice (genotypes of control mice: $Cre^{-}$, $Cre^{+/+}$, $Cre^{fl/+}$), n = 3.

**(k)** Immunofluorescent analysis of F4/80(+)Csf1r(+) and F4/80(+)Csf1r(–) cells at the bone injury sites at 1, 3, 5 and 7 dpi.

**(l)** Percentage of F4/80(+)Csf1r(+) and F4/80(+)Csf1r(–) cells at the bone injury sites at 1, 3, 5 and 7 dpi, n = 3.

**(m- o)** TRAP staining using the ELF 97 phosphatase substrate of the bone injury sites at 7 dpi by macrophage depletion methods using Clo-lip **(m)**, macrophage-lineage cell specific deletion of the *Csf1r* gene **(n)**, and AFS98 **(o)**, n = 3. The region between the two dash lines indicates the injury site.

**(p)** Summary of Figure 1 and Fig. S1: The effects of three Mφ depletion strategies (Clo-lip, AFS98, and *Csf1r* conditional knockout) on F4/80(+)Csf1r(–), F4/80(+)Csf1r(+) cell faction, and osteoclasts, as well as bone regeneration outcomes. Scale bars with its value are shown in each image. Data are shown in mean$\pm$ S.D. Statistical significance was determined by unpaired Student's t-tests. P- values are shown in each graph (**: $p < 0.01$; ***: $p < 0.001$).

**Figure. S2 Osteodirective Mφs support the proliferation and differentiation of stromal cells.**

**(a)** Immunofluorescent analysis of Tomato(+) cells in *LepR*-Cre; tdTomato mice at the bone injury sites at 7 dpi. Dash lines indicate the cortical bone surface.

**(b)** Immunofluorescent analysis of F4/80(+) cells in *LepR*-Cre; tdTomato mice in the bone injury sites at 2, 3 and 4 dpi. Dash lines indicate the boundary between injury site and uninjured bone marrow.

**(c)** Immunofluorescent analysis of F4/80(+) cells in *LepR*-Cre; tdTomato mice in the bone injury sites at 4 dpi following treatment with Ctrl-lip and Clo-lip. The number of F4/80(+) cells (left) and Tomato(+) cells (right) at the bone injury sites at 4 dpi treated with Ctrl-lip and Clo-lip,.n = 3. Dash lines indicate the boundary between injury site and uninjured bone marrow.

**(d)** Immunofluorescent analysis of Ki67(+) cells in *LepR*-Cre tdTomato mice at the bone injury site at 4 dpi and in the uninjured bone marrow area of the diaphysis following Ctrl-lip and Clo-lip treatment.

**(e)** Percentage of Ki67(+) *LepR*-Cre-labeled Tomato(+) cells among all Tomato(+) cells at the bone injury sites and in the uninjured area at 4 dpi following Ctrl-lip and Clo-lip treatment, n = 3.

**(f)** Quantitative RT-PCR analyses of *Sp7* mRNA expression in bone tissues from bone injury sites (4 dpi) and from control uninjured region of contralateral tibiae following Ctrl-lip and Clo-lip treatment, n = 4.

Scale bars: 1 mm in (a), 200 µm in (b), (c) and (f). Data are shown in mean± S.D. Statistical significance was determined by unpaired Student's t-tests. P- values are shown in each graph.

**Figure. S3 Presentative gene expression pattern in bone marrow F4/80(+) and Csf1r(+) cells.**

**(a)** Heatmap pf top5 marker genes that were differentially expressed in each cluster.

**(b)** Expression patterns of osteogenic factor encoding genes *Bmp2* and *Osm* (encoding Oncostatin M) in the UMAP visualization. The dashed line enclosed macrophage-lineage clusters.

**Figure. S4 The source of activin A production.**

**(a)** Heatmap of representative pseudotime-dependent genes from single-cell RNA-seq analyses of *Cxcl12*-CreERT2; tdTomato (+) cells isolated from femurs at 14 days after bone marrow ablation (GSE136973).

**(b)** UMAP visualization of 6 clusters using Seurat, highlighting three major classes of osteoblastic cells.

**(c)** Expression patterns of *Inhba* in the UMAP visualization.

**(d, e)** Quantitative RT-PCR analyses of *Acvr2a* and *Acvr2b* mRNA expression in bone tissues from bone injury sites at 0, 2, and 4 dpi, (n = 3 - 4 mice per each group).

**Figure. S5 Wnt inhibtors gene expression in bone repair.**

**(a)** Quantitative RT-PCR analyses of Wnt inhibitors including *Dkk1* to *4*, *Sfrp1* to *5* and *Sost* mRNA expression in bone tissues from bone injury sites (4 dpi) and uninjured region of contralateral tibiae (ctrl at 4dpi). n = 3 mice per each group.

**(b)** Quantitative RT-PCR analyses of *Sost* mRNA expression in bone tissues from bone injury sites (4 dpi) and uninjured region of contralateral tibiae (ctrl at 4dpi) treated with Ctrl-lip and Clo-lip (n = 4 mice per each group).

**Figure. S6 Wnt4 overexpression induced osteoblastogenesis in C3H10T1/2 cells.**

Detection of ALP activity of C3H10T1/2 cells after transfection by eGFP, mWnt4 and mWnt3a for 24 hours, and cultured in 96-well-plate for 7 days.

## Csf1r⁻ macrophages govern the second wave of neutrophils for bone repair

Zhifeng He[1]†, Linan Shi[2]†, Yuko Nakamichi[1], Kohei Murakami[3], Toshihide Mizoguchi[4], Yuki Matsushita[5], Hirotaka Matsumoto[6], Shinichirou Ito[7], Shunsuke Uehara[8], Rina Iwamoto[1], Takumi Takahashi[1], Zizhao Tian[9], Toru Hiraga[10], Ruoxuan Li[1], Masataka Kasahara[7], Kazuo Okamoto[11], Hiroshi Takayanagi[12], Martin M Matzuk[13], Nobuyuki Udagawa[8], Yasuhiro Kobayashi[1]*

[1]Department of Hard Tissue Research, Institute for Oral Science, Matsumoto Dental University

[2]Department of Stomatology, Affiliated Hangzhou First People' s Hospital, School of Medicine, Westlake University,

[3]Department of Veterinary Immunology, Okayama University of Science,

[4]Institute for Oral Science, Tokyo Dental College,

[5]Department of Skeletal Development and Regenerative Biology, Nagasaki University Graduate School of Biomedical Sciences,

[6]School of Information and Data Science, Nagasaki University,

[7]Department of Pharmacology, Tokyo Dental College,

[8]Department of Biochemistry, Matsumoto Dental University,

[9]Department of Biomedical Sciences, City University of Hong Kong,

[10]Department of Histology and Cell Biology, Matsumoto Dental University,

[11]Cancer Research Institute, Division of Immune Environment Dynamics, Kanazawa University,

[12]Department of Immunology, Graduate School of Medicine, The University of Tokyo,

[13]Department of Pathology& Immunology and Center for Drug Discovery, Baylor College of Medicine,

†These authors contributed equally to this work.

*Corresponding author

E-mail address: yasuhiro.kobayashi@mdu.ac.jp

1780 Hiro-oka Gobara, Shiojiri, Nagano 399-0781, Japan

Tel: +81-263-51-2238, Fax: +81-263-51-2223

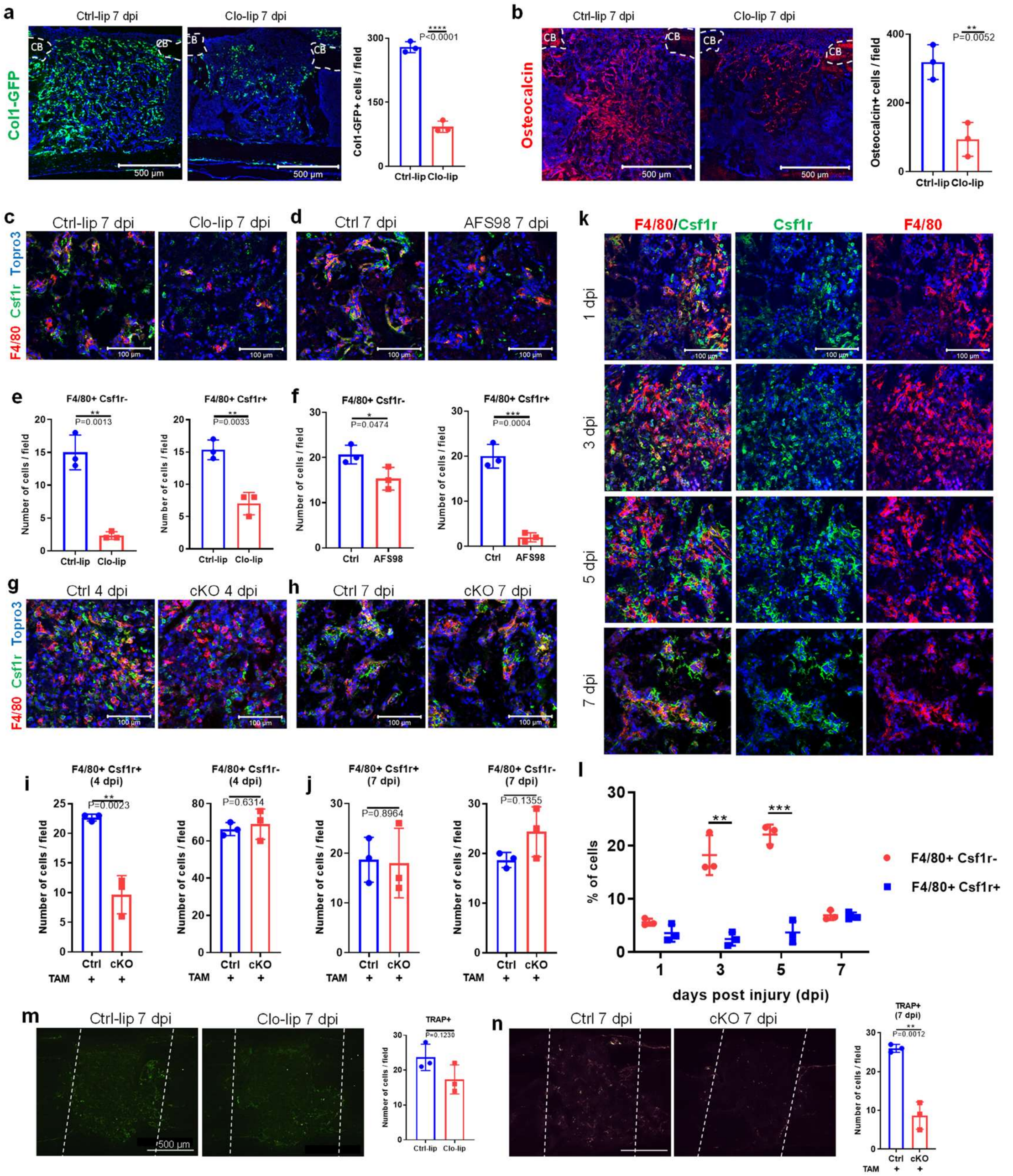

| Measurements \ Methods | Clo-lip | AFS98 | Csf1rMerCreMer Csf1r flox/flox |
|---|---|---|---|
| F4/80+ Csf1r- | ↓ | ↘ | → |
| F4/80+ Csf1r+ | ↘ | ↓ | ↘ |
| Trap+ Osteoclast | → | ↓ | ↓ |
| Regeneration conditions (7 dpi) | Significantly delayed | No difference | No difference |

**(a)** Immunofluorescent analysis of *Col1*-GFP(+) cells at the bone injury site at 7 dpi, n = 3.

**(b)** Immunofluorescent analysis of osteocalcin(+) cells at the bone injury site at 7 dpi, n = 3.

**(c- f)** Immunofluorescent analysis of F4/80(+)Csf1r(+) and F4/80(+)Csf1r(–) cells at the bone injury sites at 7 dpi from Ctrl-lip or Clo-lip-treated (**c, e**), and AFS98 or saline-treated mice (**d, f**), n = 3.

**(g- j)** Immunofluorescent analysis of F4/80(+)Csf1r(+) and F4/80(+)Csf1r(–) cells at the bone injury site at 4 and 7 dpi in *Csf1r*MerCreMer; *Csf1r* fl/fl and control mice (genotypes of control mice: $Cre^{-}$, $Cre^{+/+}$, $Cre^{fl/+}$), n = 3.

**(k)** Immunofluorescent analysis of F4/80(+)Csf1r(+) and F4/80(+)Csf1r(–) cells at the bone injury sites at 1, 3, 5 and 7 dpi.

**(l)** Percentage of F4/80(+)Csf1r(+) and F4/80(+)Csf1r(–) cells at the bone injury sites at 1, 3, 5 and 7 dpi, n = 3.

**(m- o)** TRAP staining using the ELF 97 phosphatase substrate of the bone injury sites at 7 dpi by macrophage depletion methods using Clo-lip **(m)**, macrophage-lineage cell specific deletion of the *Csf1r* gene **(n)**, and AFS98 **(o)**, n = 3. The region between the two dash lines indicates the injury site.

**(p)** Summary of Figure 1 and Fig. S1: The effects of three Mφ depletion strategies (Clo-lip, AFS98, and *Csf1r* conditional knockout) on F4/80(+)Csf1r(–), F4/80(+)Csf1r(+) cell faction, and osteoclasts, as well as bone regeneration outcomes. Scale bars with its value are shown in each image. Data are shown in mean± S.D. Statistical significance was determined by unpaired Student's t-tests. P- values are shown in each graph (**: $p < 0.01$; ***: $p < 0.001$).

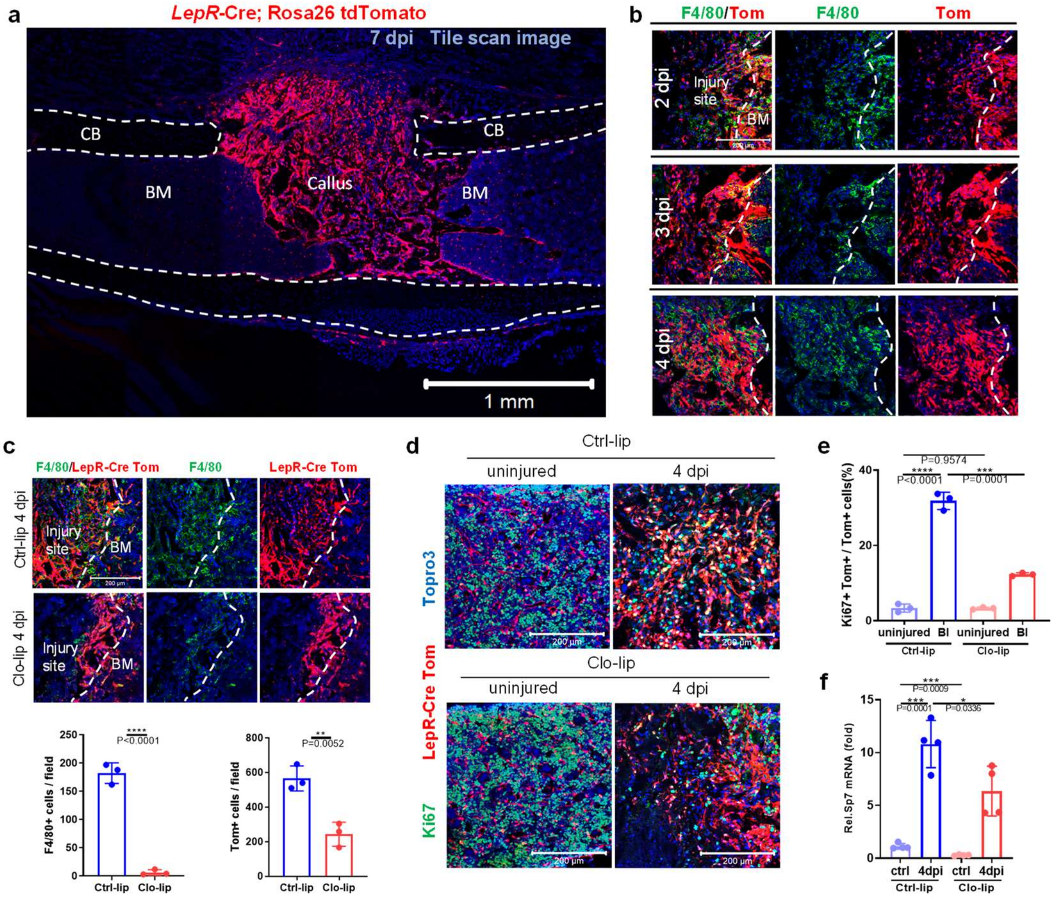

a
LepR-Cre; Rosa26 tdTomato
7 dpi Tile scan image
CB
CB
BM
Callus
BM
1 mm
b
F4/80/Tom
F4/80
Tom
2 dpi
Injury site
BM
3 dpi
4 dpi
c
F4/80/LepR-Cre Tom
F4/80
LepR-Cre Tom
Ctrl-lip 4 dpi
Injury site
BM
Clo-lip 4 dpi
Injury site
BM
F4/80+ cells / field
Ctrl-lip
Clo-lip
P<0.0001
Tom+ cells / field
P=0.0052
d
Ctrl-lip
uninjured
4 dpi
Clo-lip
uninjured
4 dpi
Topro3
LepR-Cre Tom
Ki67
200 µm
e
Ki67+ Tom+ / Tom+ cells(%)
P=0.9574
P<0.0001
P=0.0001
uninjured
BI
uninjured
BI
Ctrl-lip
Clo-lip
f
Rel.Sp7 mRNA (fold)
P=0.0009
P=0.0001
P=0.0336
ctrl
4dpi
ctrl
4dpi
Ctrl-lip
Clo-lip

61

**(a)** Immunofluorescent analysis of Tomato(+) cells in *LepR*-Cre; tdTomato mice at the bone injury sites at 7 dpi. Dash lines indicate the cortical bone surface.

**(b)** Immunofluorescent analysis of F4/80(+) cells in *LepR*-Cre; tdTomato mice in the bone injury sites at 2, 3 and 4 dpi. Dash lines indicate the boundary between injury site and uninjured bone marrow.

**(c)** Immunofluorescent analysis of F4/80(+) cells in *LepR*-Cre; tdTomato mice in the bone injury sites at 4 dpi following treatment with Ctrl-lip and Clo-lip. The number of F4/80(+) cells (left) and Tomato(+) cells (right) at the bone injury sites at 4 dpi treated with Ctrl-lip and Clo-lip,.n = 3. Dash lines indicate the boundary between injury site and uninjured bone marrow.

**(d)** Immunofluorescent analysis of Ki67(+) cells in *LepR*-Cre tdTomato mice at the bone injury site at 4 dpi and in the uninjured bone marrow area of the diaphysis following Ctrl-lip and Clo-lip treatment.

**(e)** Percentage of Ki67(+) *LepR*-Cre-labeled Tomato(+) cells among all Tomato(+) cells at the bone injury sites and in the uninjured area at 4 dpi following Ctrl-lip and Clo-lip treatment, n = 3.

**(f)** Quantitative RT-PCR analyses of *Sp7* mRNA expression in bone tissues from bone injury sites (4 dpi) and from control uninjured region of contralateral tibiae following Ctrl-lip and Clo-lip treatment, n = 4.

Scale bars: 1 mm in (a), 200 μm in (b), (c) and (f). Data are shown in mean± S.D. Statistical significance was determined by unpaired Student's t-tests. P- values are shown in each graph.

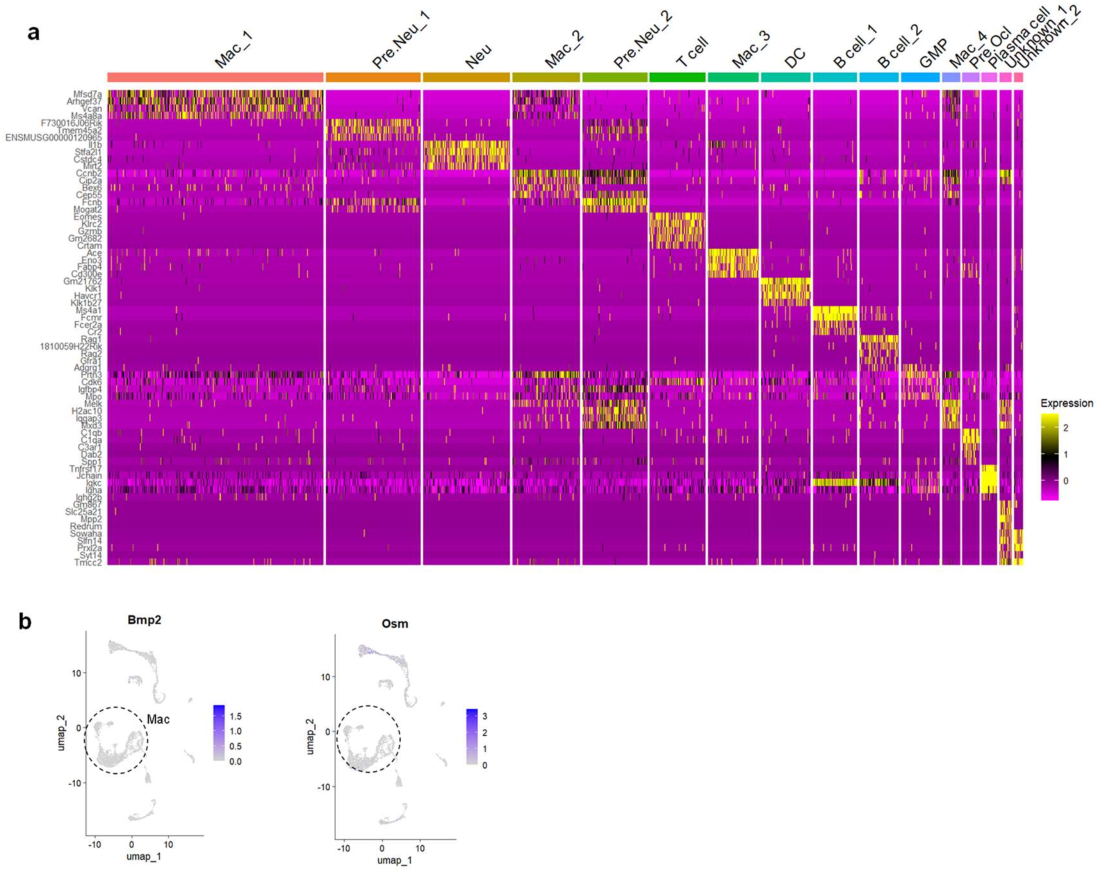

a
Mac_1
Pre.Neu_1
Neu
Mac_2
Pre.Neu_2
T cell
Mac_3
DC
B cell_1
B cell_2
GMP
Mac_4
Pre.Ocl
Plasma cell
Unknown_1
Unknown_2
Expression
2
1
0
b
Bmp2
Mac
umap_2
umap_1
1.5
1.0
0.5
0.0
Osm
3
2
1
0
10
0
-10

**(a)** Heatmap pf top5 marker genes that were differentially expressed in each cluster.

**(b)** Expression patterns of osteogenic factor encoding genes *Bmp2* and *Osm* (encoding Oncostatin M) in the UMAP visualization. The dashed line enclosed macrophage-lineage clusters.

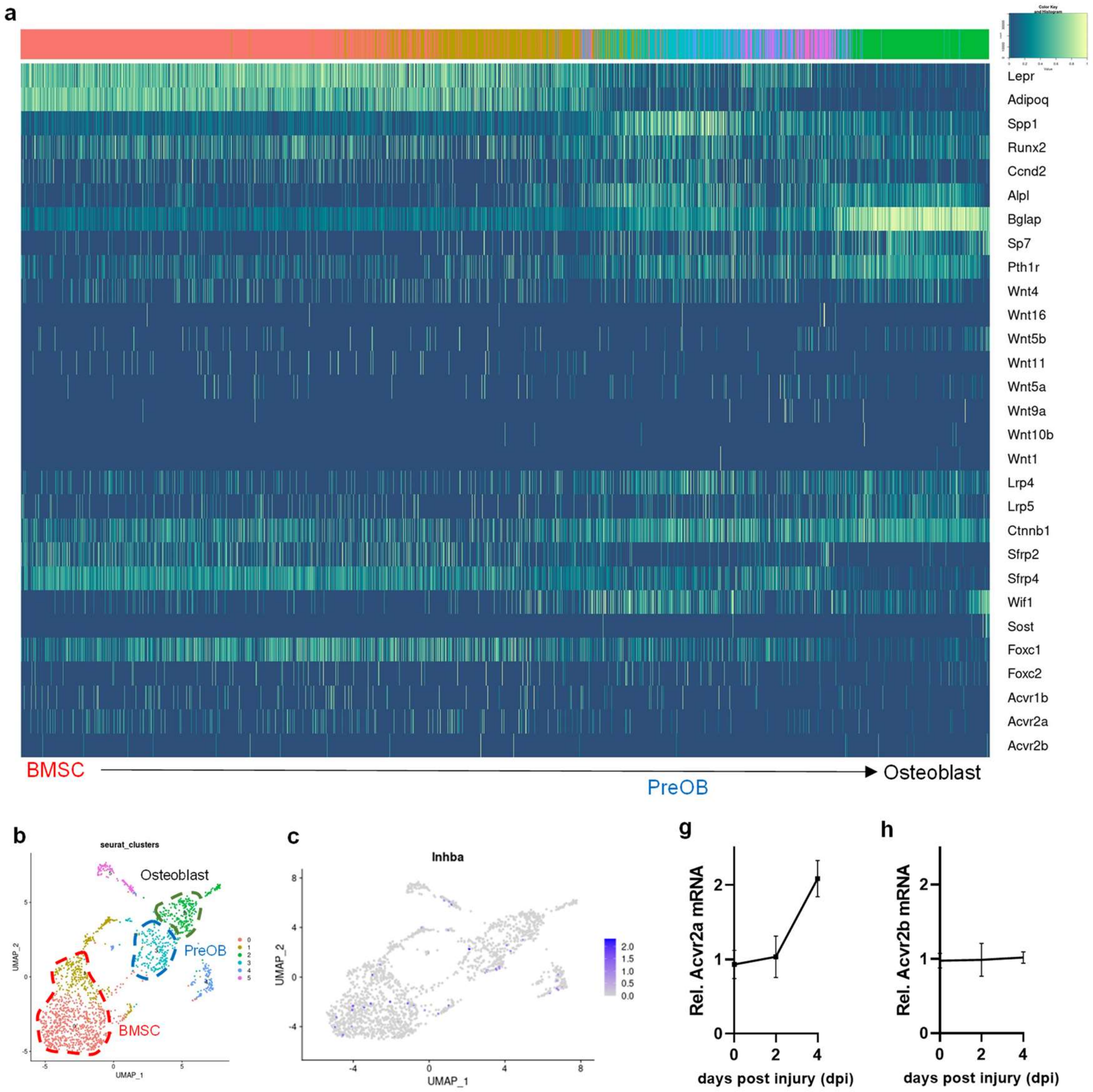

a
Lepr
Adipoq
Spp1
Runx2
Ccnd2
Alpl
Bglap
Sp7
Pth1r
Wnt4
Wnt16
Wnt5b
Wnt11
Wnt5a
Wnt9a
Wnt10b
Wnt1
Lrp4
Lrp5
Ctnnb1
Sfrp2
Sfrp4
Wif1
Sost
Foxc1
Foxc2
Acvr1b
Acvr2a
Acvr2b
BMSC
Osteoblast
PreOB
b
seurat_clusters
Osteoblast
PreOB
BMSC
UMAP_1
UMAP_2
c
Inhba
UMAP_1
UMAP_2
g
Rel. Acvr2a mRNA
days post injury (dpi)
h
Rel. Acvr2b mRNA
days post injury (dpi)

118

119

**Figure. S4 The source of activin A production.**

**(a)** Heatmap of representative pseudotime-dependent genes from single-cell RNA-seq analyses of *Cxcl12*-CreERT2; tdTomato (+) cells isolated from femurs at 14 days after bone marrow ablation (GSE136973).

**(b)** UMAP visualization of 6 clusters using Seurat, highlighting three major classes of osteoblastic cells.

**(c)** Expression patterns of *Inhba* in the UMAP visualization.

**(d, e)** Quantitative RT-PCR analyses of *Acvr2a* and *Acvr2b* mRNA expression in bone tissues from bone injury sites at 0, 2, and 4 dpi, (n = 3 - 4 mice per each group).

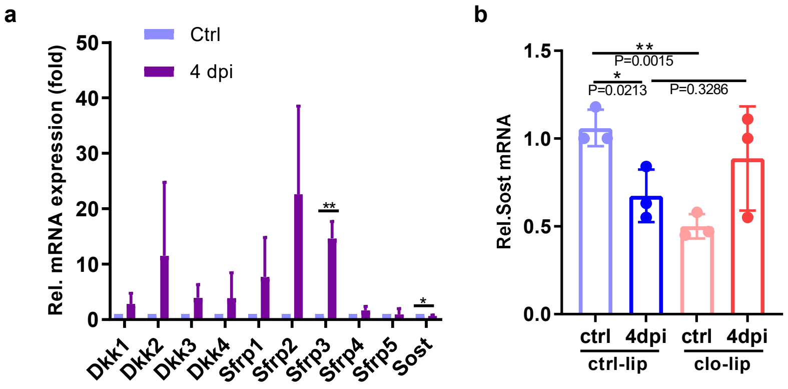

a
Ctrl
4 dpi
Rel. mRNA expression (fold)
50
40
30
20
10
0
**
*
Dkk1
Dkk2
Dkk3
Dkk4
Sfrp1
Sfrp2
Sfrp3
Sfrp4
Sfrp5
Sost
b
Rel.Sost mRNA
1.5
1.0
0.5
0.0
**
P=0.0015
*
P=0.0213
P=0.3286
ctrl
4dpi
ctrl
4dpi
ctrl-lip
clo-lip

**Figure. S5 Wnt inhibtors gene expression in bone repair.**

**(a)** Quantitative RT-PCR analyses of Wnt inhibitors including *Dkk1* to *4*, *Sfrp1* to *5* and *Sost* mRNA expression in bone tissues from bone injury sites (4 dpi) and uninjured region of contralateral tibiae (ctrl at 4dpi). n = 3 mice per each group.

**(b)** Quantitative RT-PCR analyses of *Sost* mRNA expression in bone tissues from bone injury sites (4 dpi) and uninjured region of contralateral tibiae (ctrl at 4dpi) treated with Ctrl-lip and Clo-lip (n = 4 mice per each group).

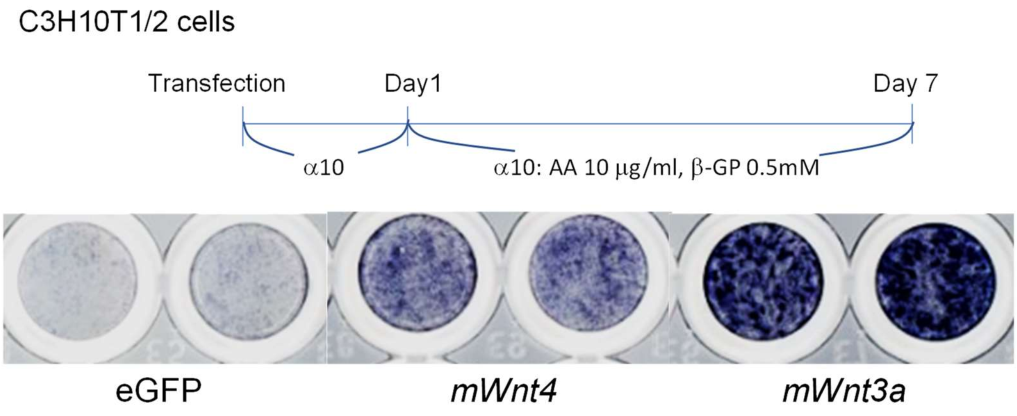

C3H10T1/2 cells
Transfection
Day1
Day 7
α10
α10: AA 10 μg/ml, β-GP 0.5mM
eGFP
mWnt4
mWnt3a

Detection of ALP activity of C3H10T1/2 cells after transfection by eGFP, mWnt4 and mWnt3a for 24 hours, and cultured in 96-well-plate for 7 days.

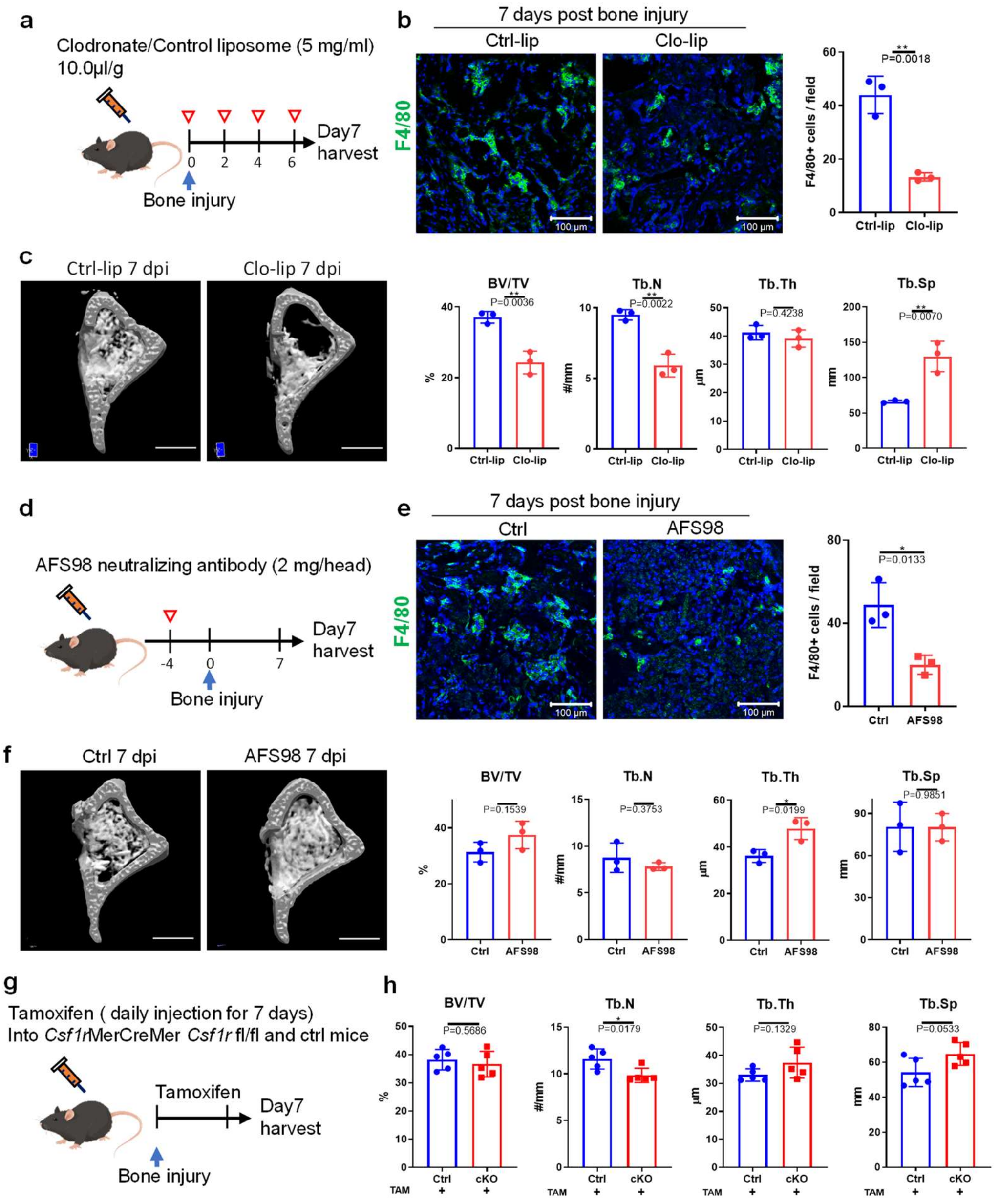

a
Clodronate/Control liposome (5 mg/ml)
10.0µl/g
Day7
harvest
Bone injury
b
7 days post bone injury
Ctrl-lip
Clo-lip
F4/80
100 µm
F4/80+ cells / field
P=0.0018
c
Ctrl-lip 7 dpi
Clo-lip 7 dpi
BV/TV
P=0.0036
Tb.N
P=0.0022
Tb.Th
P=0.4238
Tb.Sp
P=0.0070
d
AFS98 neutralizing antibody (2 mg/head)
Day7
harvest
Bone injury
e
7 days post bone injury
Ctrl
AFS98
F4/80
100 µm
F4/80+ cells / field
P=0.0133
f
Ctrl 7 dpi
AFS98 7 dpi
BV/TV
P=0.1539
Tb.N
P=0.3753
Tb.Th
P=0.0199
Tb.Sp
P=0.9851
g
Tamoxifen ( daily injection for 7 days)
Into Csf1rMerCreMer Csf1r fl/fl and ctrl mice
Tamoxifen
Day7
harvest
Bone injury
h
BV/TV
P=0.5686
Tb.N
P=0.0179
Tb.Th
P=0.1329
Tb.Sp
P=0.0533
Ctrl
cKO
TAM

**Figure. 1**

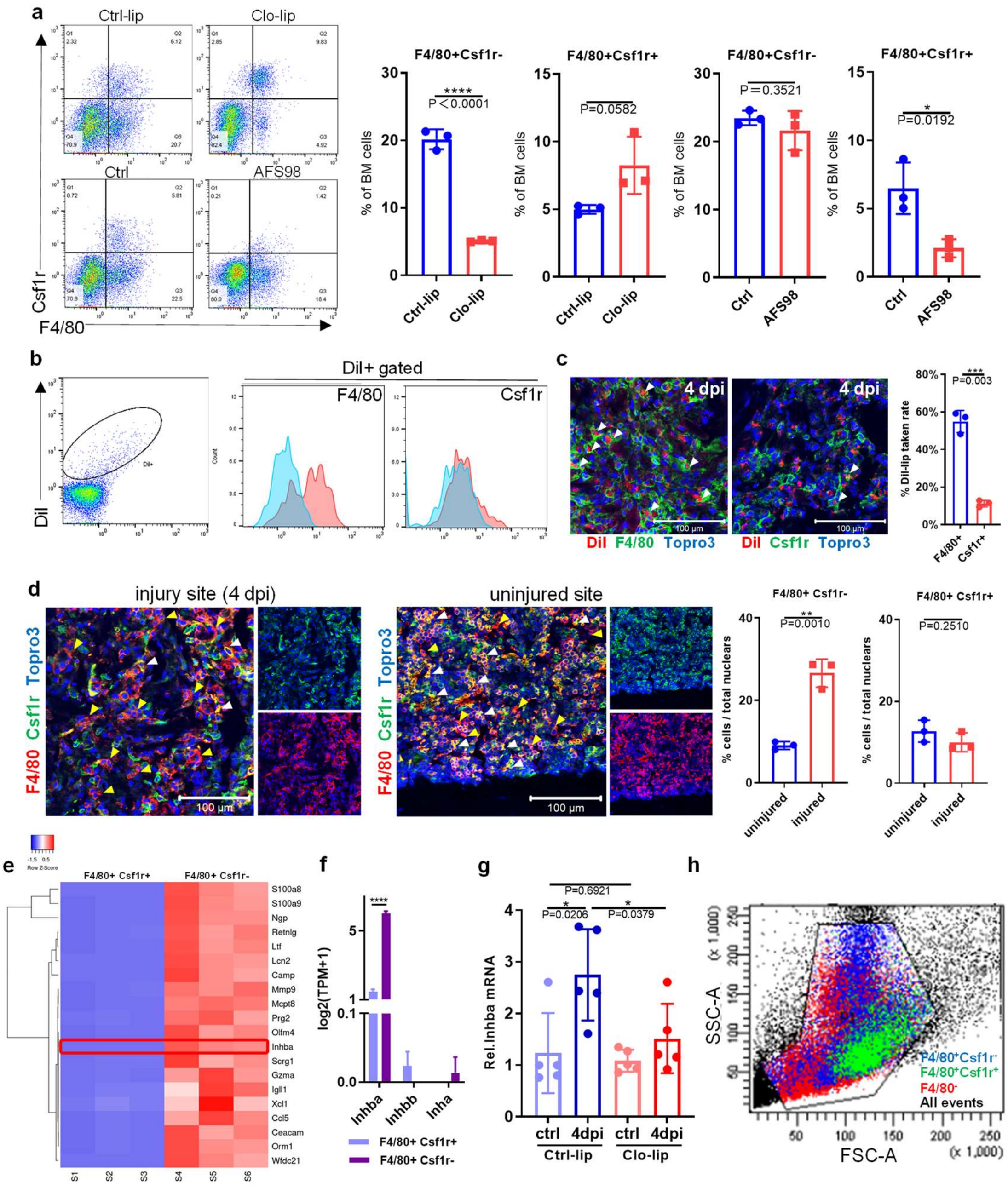

3

4 **Figure. 2**

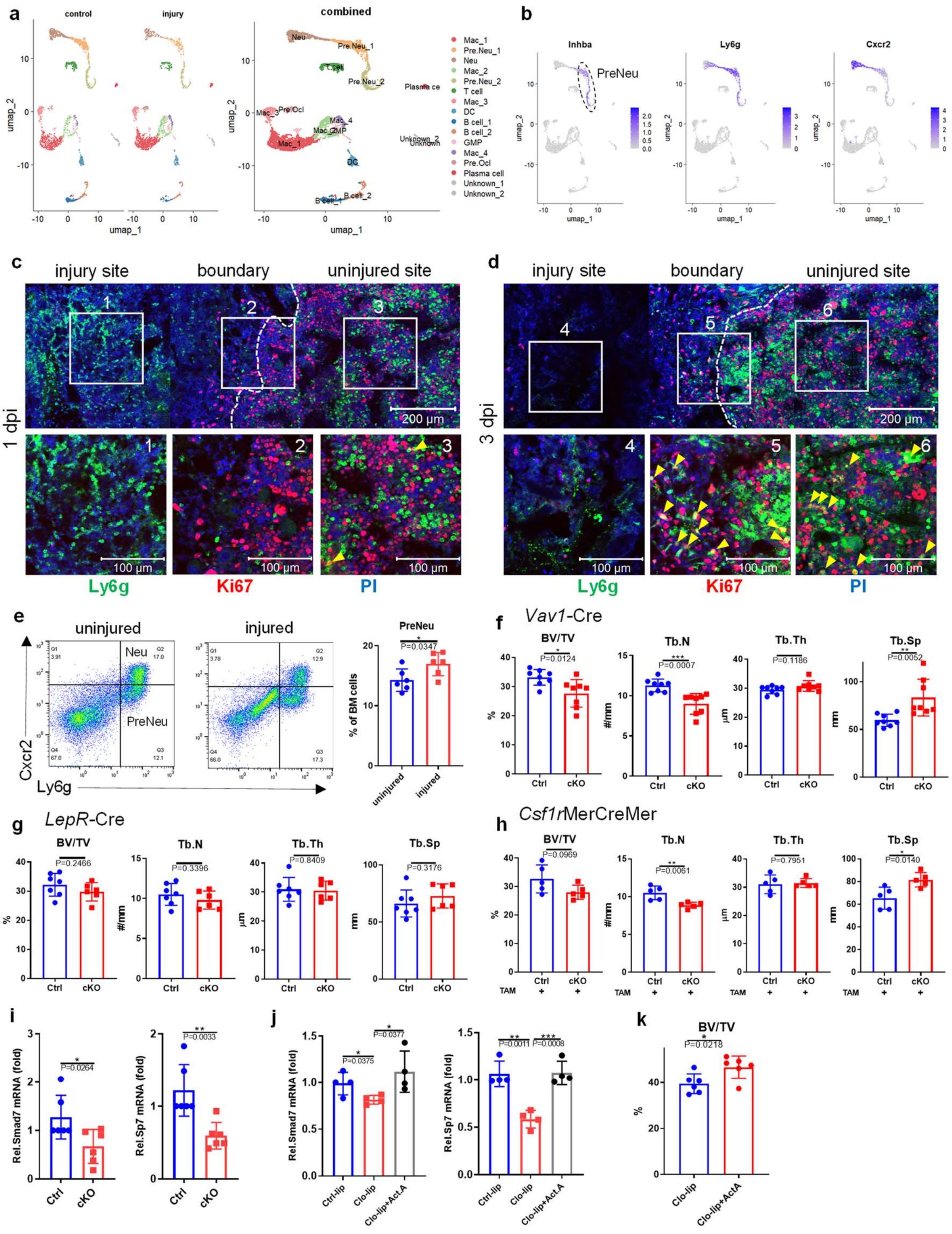

a
control
injury
combined
Mac_1
Pre.Neu_1
Neu
Mac_2
Pre.Neu_2
T cell
Mac_3
DC
B cell_1
B cell_2
GMP
Mac_4
Pre.Ocl
Plasma cell
Unknown_1
Unknown_2
b
Inhba
PreNeu
Ly6g
Cxcr2
c
injury site
boundary
uninjured site
1 dpi
200 µm
100 µm
Ly6g
Ki67
PI
d
3 dpi
e
uninjured
injured
Neu
PreNeu
Cxcr2
Ly6g
PreNeu
P=0.0347
% of BM cells
f
Vav1-Cre
BV/TV
P=0.0124
Tb.N
P=0.0007
Tb.Th
P=0.1186
Tb.Sp
P=0.0052
Ctrl
cKO
g
LepR-Cre
P=0.2466
P=0.3396
P=0.8409
P=0.3176
h
Csf1rMerCreMer
P=0.0969
P=0.0061
P=0.7951
P=0.0140
TAM
i
Rel.Smad7 mRNA (fold)
P=0.0264
Rel.Sp7 mRNA (fold)
P=0.0033
j
P=0.0375
P=0.0377
P=0.0011
P=0.0008
Ctrl-lip
Clo-lip
Clo-lip+Act.A
k
P=0.0218
Clo-lip+ActA

5

6 **Figure. 3**

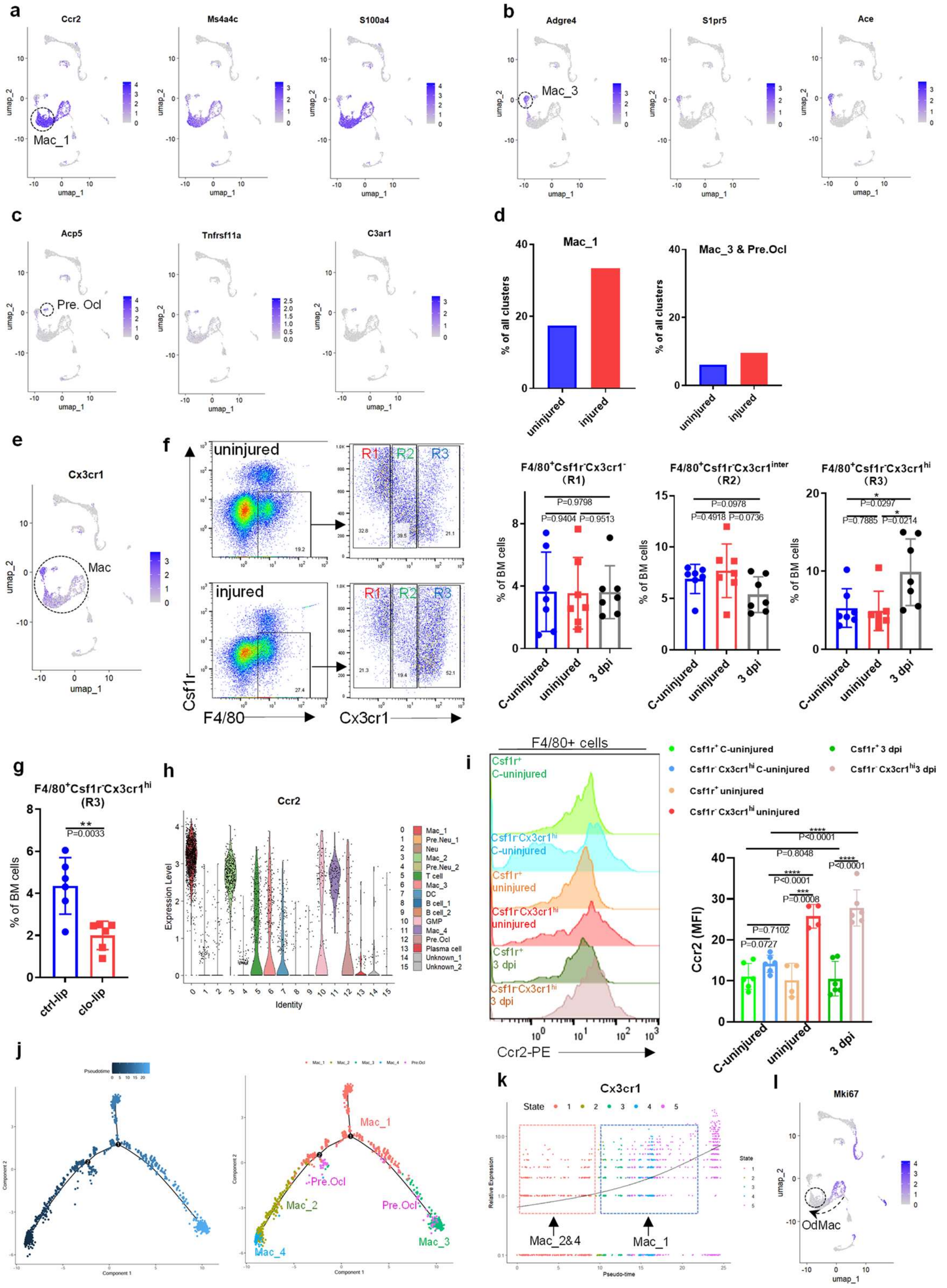

a
Ccr2
Ms4a4c
S100a4
Mac_1
b
Adgre4
S1pr5
Ace
Mac_3
c
Acp5
Tnfrsf11a
C3ar1
Pre. Ocl
d
Mac_1
Mac_3 & Pre.Ocl
% of all clusters
uninjured
injured
e
Cx3cr1
Mac
f
uninjured
injured
R1
R2
R3
Csf1r
F4/80
Cx3cr1
F4/80+Csf1r-Cx3cr1- (R1)
F4/80+Csf1r-Cx3cr1inter (R2)
F4/80+Csf1r-Cx3cr1hi (R3)
% of BM cells
C-uninjured
uninjured
3 dpi
g
F4/80+Csf1r-Cx3cr1hi (R3)
P=0.0033
ctrl-lip
clo-lip
h
Ccr2
Expression Level
Identity
i
F4/80+ cells
Ccr2-PE
Ccr2 (MFI)
j
Pseudotime
Component 1
Component 2
Mac_1
Mac_2
Mac_3
Mac_4
Pre.Ocl
k
Cx3cr1
State
Relative Expression
Pseudo-time
Mac_2&4
Mac_1
l
Mki67
OdMac

**Figure. 4**

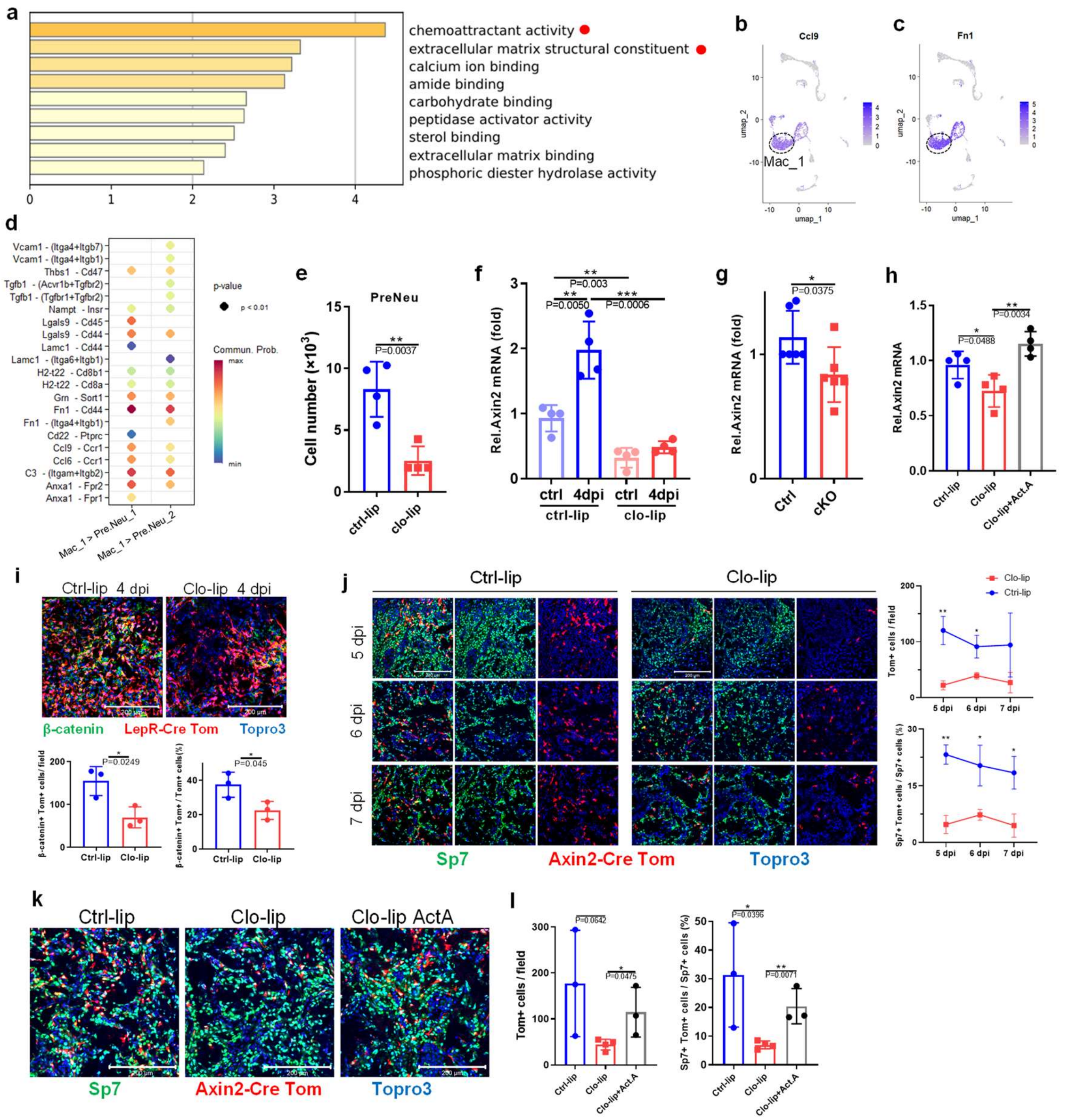

chemoattractant activity
extracellular matrix structural constituent
calcium ion binding
amide binding
carbohydrate binding
peptidase activator activity
sterol binding
extracellular matrix binding
phosphoric diester hydrolase activity
Ccl9
Fn1
Mac_1
PreNeu
Cell number (×10³)
ctrl-lip
clo-lip
Rel.Axin2 mRNA (fold)
Ctrl
cKO
Rel.Axin2 mRNA
Ctrl-lip
Clo-lip
Clo-lip+Act.A
Ctrl-lip 4 dpi
Clo-lip 4 dpi
β-catenin
LepR-Cre Tom
Topro3
5 dpi
6 dpi
7 dpi
Sp7
Axin2-Cre Tom
Clo-lip ActA

9

10 **Figure. 5**

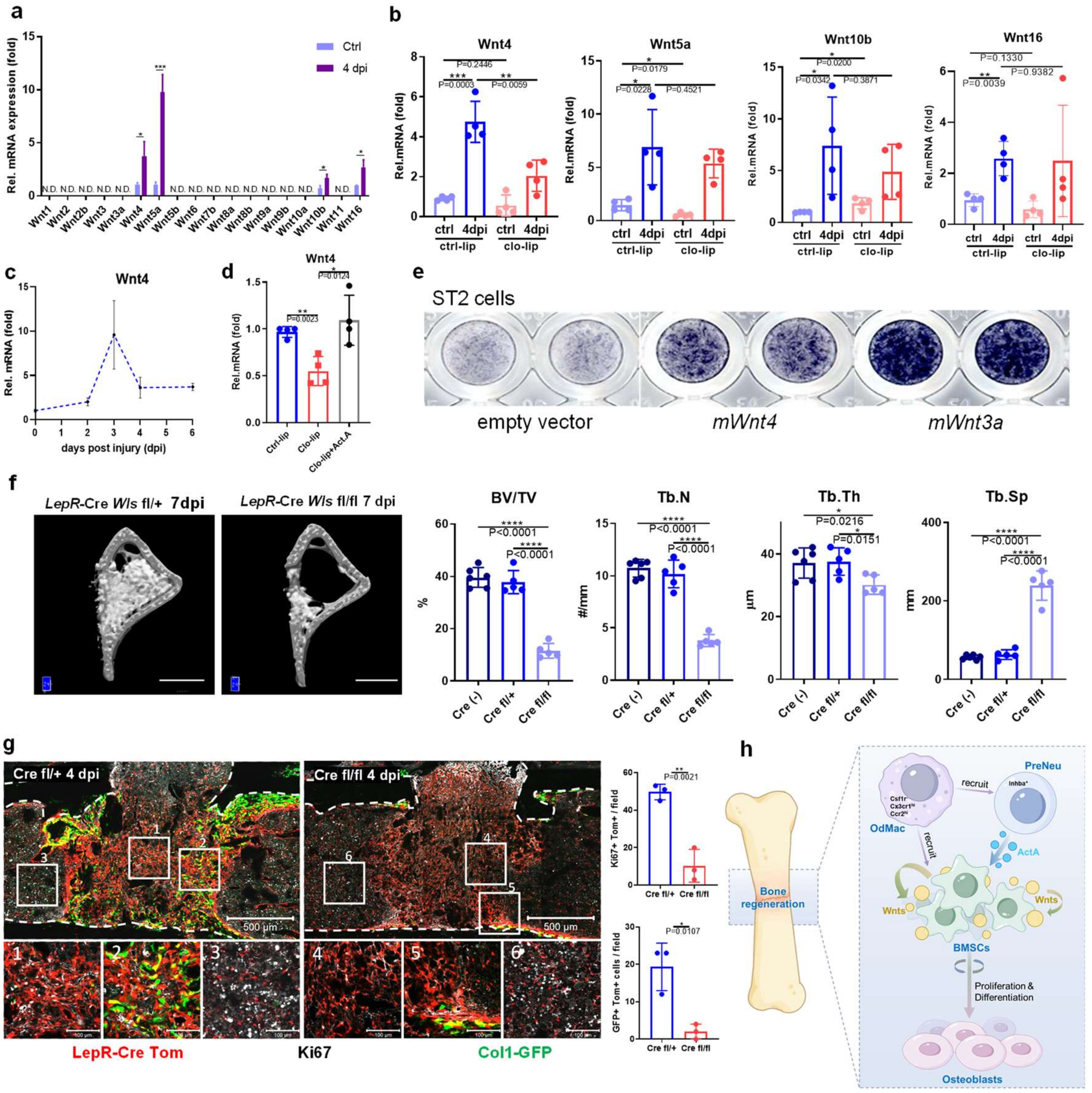

a
Rel. mRNA expression (fold)
Ctrl
4 dpi
N.D.
Wnt1
Wnt2
Wnt2b
Wnt3
Wnt3a
Wnt4
Wnt5a
Wnt5b
Wnt6
Wnt7b
Wnt8a
Wnt8b
Wnt9a
Wnt9b
Wnt10a
Wnt10b
Wnt11
Wnt16
b
Wnt4
Wnt5a
Wnt10b
Wnt16
Rel.mRNA (fold)
P=0.2446
P=0.0003
P=0.0059
P=0.0179
P=0.0228
P=0.4521
P=0.0200
P=0.0342
P=0.3871
P=0.1330
P=0.0039
P=0.9382
ctrl
4dpi
ctrl-lip
clo-lip
c
Wnt4
Rel. mRNA (fold)
days post injury (dpi)
d
Wnt4
P=0.0023
P=0.0124
Ctrl-lip
Clo-lip
Clo-lip+ActA
e
ST2 cells
empty vector
mWnt4
mWnt3a
f
LepR-Cre Wls fl/+ 7dpi
LepR-Cre Wls fl/fl 7 dpi
BV/TV
Tb.N
Tb.Th
Tb.Sp
P<0.0001
P=0.0216
P=0.0151
Cre (-)
Cre fl/+
Cre fl/fl
g
Cre fl/+ 4 dpi
Cre fl/fl 4 dpi
500 μm
LepR-Cre Tom
Ki67
Col1-GFP
Ki67+ Tom+ / field
P=0.0021
GFP+ Tom+ cells / field
P=0.0107
h
Bone regeneration
OdMac
recruit
PreNeu
ActA
Wnts
BMSCs
Proliferation & Differentiation
Osteoblasts

**Figure. 6**

## Supplementary Files

This is a list of supplementary files associated with this preprint. Click to download.

- Heetal.05192025SFig.pdf
